# Turnip Mosaic Potyvirus Probably First Spread to Eurasian Brassica Crops from Wild Orchids about 1000 Years Ago

**DOI:** 10.1371/journal.pone.0055336

**Published:** 2013-02-06

**Authors:** Huy D. Nguyen, Yasuhiro Tomitaka, Simon Y. W. Ho, Sebastián Duchêne, Heinrich-Josef Vetten, Dietrich Lesemann, John A. Walsh, Adrian J. Gibbs, Kazusato Ohshima

**Affiliations:** 1 Laboratory of Plant Virology, Faculty of Agriculture, Saga University, Saga, Japan; 2 The United Graduate School of Agricultural Sciences, Kagoshima University, Kagoshima, Japan; 3 School of Biological Sciences, University of Sydney, Sydney, Australia; 4 Julius Kuehn Institute, Federal Research Centre for Cultivated Plants (JKI), Institute of Epidemiology and Pathogen Diagnostics, Braunschweig, Germany; 5 Life Sciences, University of Warwick, Wellesbourne, Warwick, United Kingdom; 6 Emeritus Faculty, Australian National University, Canberra, Australia; University of California, Riverside, United States of America

## Abstract

Turnip mosaic potyvirus (TuMV) is probably the most widespread and damaging virus that infects cultivated brassicas worldwide. Previous work has indicated that the virus originated in western Eurasia, with all of its closest relatives being viruses of monocotyledonous plants. Here we report that we have identified a sister lineage of TuMV-like potyviruses (TuMV-OM) from European orchids. The isolates of TuMV-OM form a monophyletic sister lineage to the brassica-infecting TuMVs (TuMV-BIs), and are nested within a clade of monocotyledon-infecting viruses. Extensive host-range tests showed that all of the TuMV-OMs are biologically similar to, but distinct from, TuMV-BIs and do not readily infect brassicas. We conclude that it is more likely that TuMV evolved from a TuMV-OM-like ancestor than the reverse. We did Bayesian coalescent analyses using a combination of novel and published sequence data from four TuMV genes [helper component-proteinase protein (HC-Pro), protein 3(P3), nuclear inclusion b protein (NIb), and coat protein (CP)]. Three genes (HC-Pro, P3, and NIb), but not the CP gene, gave results indicating that the TuMV-BI viruses diverged from TuMV-OMs around 1000 years ago. Only 150 years later, the four lineages of the present global population of TuMV-BIs diverged from one another. These dates are congruent with historical records of the spread of agriculture in Western Europe. From about 1200 years ago, there was a warming of the climate, and agriculture and the human population of the region greatly increased. Farming replaced woodlands, fostering viruses and aphid vectors that could invade the crops, which included several brassica cultivars and weeds. Later, starting 500 years ago, inter-continental maritime trade probably spread the TuMV-BIs to the remainder of the world.

## Introduction

The possibility of controlling a pathogen is improved if we know when, where, and how it first became established in the host population, namely its ‘centre of emergence’. This is analogous to the ‘centre of diversity’ of crop species [1], [2]. It is valuable to identify this centre because it may still contain the pathogen and host populations most closely related to those involved in the emergence. Therefore, these populations might have been interacting with the pathogen longer than others, leading to the greatest diversity in the genes controlling that interaction. As a consequence, such populations might be useful in the design of gene-based control strategies. We have previously reported phylogeographic studies of *Turnip mosaic virus* (TuMV), which is probably the world’s most widespread and damaging virus of domesticated species of the family *Brassicaceae*, both crop and ornamental [3]–[6]. These studies clearly indicated that present-day TuMV populations came from a founder population in western Eurasia, namely Europe, Asia Minor, and the Middle East. However, the source virus, source populations and the timing of that emergence remained unknown.

TuMV is a species of the genus *Potyvirus*, one of the two largest genera of plant viruses and containing nine-tenths of the species of the family *Potyviridae* [7]. Potyviruses infect a wide range of mono- and dicotyledonous plant species [8]. They are spread by aphids in a non-persistent manner, and also in seed and infected living plant materials. They have flexuous filamentous particles 700–750 nm long, each of which contains a single copy of the genome. The genome is a single-stranded, positive-sense RNA molecule of approximately 10,000 nt with one major open reading frame (ORF) that is translated into one large polyprotein and with a small overlapping ORF [9]. The polyprotein is autocatalytically hydrolyzed into at least ten proteins [7], [8].

The world population of TuMV has probably been more thoroughly sampled and sequenced than that of any other potyvirus. Our previous studies of a worldwide collection of ca. 100 TuMV isolates [3] showed that the virus has four phylogenetic lineages. The four host types are mostly congruent with the phylogenetic groupings. Isolates from host type [(B)] occasionally infect *Brassica* plants, often latently, but not *Raphanus* plants. Isolates from host type [B] infect most *Brassica* species, giving mosaic systemic symptoms, but do not infect *Raphanus* plants. Isolates from host type [B(R)] give systemic mosaics in most *Brassica* species and occasionally infect *Raphanus* plants latently. Isolates from host type [BR] give systemic mosaic symptoms in both *Brassica* and *Raphanus* plants. The most variable of the four major TuMV clusters is the paraphyletic basal-*Brassica* (basal-B) cluster of [(B)] pathotype isolates. It includes isolates from brassicas, namely cultivated and wild species of *Brassicaceae*, and also species from other families mostly collected in Europe. The remaining isolates fall into two monophyletic sister clusters: the world-*Brassica* (world-B) cluster is the more variable and widespread cluster, and the less variable cluster of [BR] isolates has two sub-clusters, the basal-*Brassica*/*Raphanus* (basal-BR) and the Asian-*Brassica*/*Raphanus* (Asian-BR) clusters [3], [5].

TuMV is one of more than 70 potyviruses, each represented by at least one complete genomic sequence in the Genbank database. A phylogeny inferred from the polyproteins encoded by these genomes [8] revealed that there are at least 11 distinct lineages of potyviruses. One of these is the ‘TuMV group’, which comprises TuMV and at least five other species, all from monocotyledonous plants. These include *Japanese yam mosaic virus* (JYMV) [10], [11], *Narcissus yellow stripe virus* (NYSV) [12], and *Scallion mosaic virus* (ScMV) [13], all known from full genomic sequences. Sequence analyses of the coat protein have shown that the TuMV group also contains Indian narcissus potyvirus and Narcissus late season yellows potyvirus. In a genomic potyvirus phylogeny [8], TuMV is nested within this group, and all the closest relatives of the virus are from monocotyledons. This suggests that the ability to infect monocotyledons is probably an ancestral character of the TuMV group, and that TuMV’s ability to infect brassicas, dicotyledonous plants, is a recent adaptation.

A phylogeographic analysis of the entire potyvirus genus [8] indicated that the genus, like TuMV, originated in western Eurasia and/or North Africa, and probably evolved from a virus of monocotyledonous plants. All of the species of the two earliest-diverging lineages of potyviruses were first isolated from monocotyledonous plants, which were first domesticated in the same region [14], [15], as too were all species of *Rymovirus*, the close sister genus to *Potyvirus.* The basal divergence in the phylogeny of all potyviruses was estimated to have occurred around 7,250 years before present (YBP) [8], [16].

In this paper, we report that a cluster of biologically-distinct TuMV-like viruses, isolated from European orchids [17], [18], possesses the suite of characters likely to be found in the viruses from which the brassica-infecting TuMVs evolved. We also estimate that the brassica-infecting lineage first diverged from the orchid-infecting viruses around 1000 years ago.

## Materials and Methods

### Virus Isolates and Host Tests

Isolates used in the present study were mostly collected from various host plants in European countries including Belgium, Denmark, France, Germany, Greece, Hungary, Italy, Poland, Portugal, Spain, The Netherlands, and the United Kingdom. Isolates were also collected in non-European countries including the United States of America. The isolates were collected from private gardens and fields. We asked the owners for permission to collect samples from their properties. Some samples came from colleagues and these are listed in the Acknowledgements. None of the samples were from ‘endangered’ species. Details of the isolates are shown in Table S1, together with those of the isolates used in the sequence analyses and for which the complete genomic sequences have already been reported [4], [6], [19]–[30]; GenBank Accession Codes (AF394601, AF394602, and EF374098). The orchid-infecting TuMV-like viruses were isolated from *Orchis militaris, Orchis morio*, and *Orchis simia* plants growing in a collection at Celle, Germany. These isolates were collected by Vetten and Lesemann, one of authors of the present study, and details of these isolates have already been published [17]. The isolates were collected by the permission of the owners. OM isolates were also found in nine other species of Orchidaceae in the same collection, so it is uncertain which species were the original source of the TuMV-OM viruses, although all the orchids have overlapping natural distributions in eastern, central, and southern Europe.

All of the isolates were sap-inoculated to *Chenopodium quinoa* plants and serially cloned through single lesions at least three times. TuMV isolates were generally cloned by single lesion isolations in the earlier [3]–[6] and present studies because of the high frequency of mixed infections in the field, not only with other viruses but also other isolates of the same virus. Thus, biological cloning is mandatory when attempting to analyse recombination events and the genetic structure of populations. They were propagated in *Brassica rapa* cv. Hakatasuwari or *Nicotiana benthamiana* plants. Plants infected systemically with each of the TuMV isolates were homogenized in 0.01 M potassium phosphate buffer (pH 7.0) and mechanically inoculated on to young plants of *B. rapa* cv. Hakatasuwari, and *Raphanus sativus* cvs Taibyo-sobutori and Akimasari. Inoculated plants were kept for at least four weeks in a glasshouse at 25°C. The isolates collected from *Orchis*, along with some other isolates, were also tested for host reactions using plants from a broader range of species.

### Viral RNA and Sequencing

Viral RNAs were extracted from TuMV-infected *B. rapa* and *N. benthamiana* leaves using Isogen (Nippon Gene). The RNAs were reverse-transcribed and amplified using high-fidelity Platinum *Pfx* DNA polymerase (Invitrogen). cDNAs were separated by electrophoresis in agarose gels and purified using a QIAquick Gel Extraction kit (Qiagen). Sequences from each isolate were determined using at least four overlapping independent RT-PCR products to cover the complete genome. The sequences of the RT-PCR products or cloned fragments of adjacent regions of the genome overlapped by at least 200 nt to ensure that they were from the same genome and were not from different components of a genome mixture. Each RT-PCR product was sequenced by primer walking in both directions using a BigDye Terminator v3.1 Cycle Sequencing Ready Reaction kit (Applied Biosystems) and an Applied Biosystems Genetic Analyser DNA model 310. Ambiguous nucleotides in any sequence were checked in sequences obtained from at least five other independent plasmids as described in the earlier studies [3]–[6]. Sequence data were assembled using BioEdit version 5.0.9 [31]. The similarity of nucleotide sequences between TuMV isolates and group viruses was determined using SIMPLOT version 3.5.1 [32] with a window length of 200 and step size of 20.

### Recombination Analyses

The genomic sequences of the 155 isolates were used for evolutionary analyses. Two genomic sequences of JYMV [10], [11], one of ScMV [12], and one of NYSV [13] were used as outgroups because BLAST searches had shown them to be most closely and consistently related to those of TuMV. TuMV Protein 1 (P1) genes were more closely related to those of JYMV than to those of ScMV, whereas for some intergenic regions between helper component-proteinase protein (HC-Pro) and nuclear inclusion b protein (NIb) the converse was true. The TuMV coat protein (CP) gene was most closely related to that of NYSV. Therefore, we used CLUSTAL X2 [33] to align all 155 P1 gene sequences with those of two JYMV isolates, the CP sequences with that of NYSV, and the remaining sequences with those of JYMV and ScMV. To ensure that the alignments were in frame, all genes were aligned via the corresponding amino acid sequences using CLUSTAL X2 with TRANSALIGN (kindly supplied by Georg Weiller, Australian National University) and were then reassembled to form complete ORF sequences of 9321 nt. The aligned sequences of the 5′ and 3′ non-coding regions were then added.

We investigated recombination in the genomic sequences using the split-decomposition method implemented in SPLITSTREE version 4.11.3 [34]. As no single algorithm conclusively identifies all putative recombination breakpoints, we used a combination of those in the RDP package [35], namely GENECONV [36], BOOTSCAN [37], MAXCHI [38], CHIMAERA [39], and SISCAN programs [40] implemented in RDP3 [41] and original PHYLPRO version 1 [42], SISCAN version 2 [40], and SISCAN M (kindly provided by M. J. Gibbs and J. S. Armstrong) programs. These analyses were done using default settings for the different detection programs and a Bonferroni-corrected *P*-value cut-off of 0.05 or 0.01 to search for recombination events supported by three different methods with an associated *P*-value of >1.0×10^−6^.

We checked each identified recombinant by estimating the phylogeny of the recombinant parts to verify the parent/daughter assignment made by RDP3. Next, all sequences that had been identified as likely recombinants, together with all sequences used in this study, were checked again using original PHYLPRO and SISCAN programs. These analyses were done not only with all nucleotide sites, but also with synonymous and non-synonymous sites separately. We checked 100- and 50-nt slices of all sequences for evidence of recombination using these programs. These analyses also assessed which non-recombinant sequences had regions that were closest to those of the recombinant sequences and hence indicated the likely lineages that provided those regions of the recombinant genomes. For simplicity, we called these the ‘parental isolates’ of recombinants, although in reality they were merely those that were most closely related to the parental isolates among those that we were analysing. Finally, TuMV sequences were also aligned without outgroup sequences, and directly checked for evidence of recombination using the programs.

### Phylogenetic Analyses

To estimate the phylogenetic relationships among the TuMV isolates and the outgroups, we analysed the sequences using maximum likelihood PhyML version 3 [43]. Many recombinant genomes had been identified in previous studies [4] and were discarded for our timescale analyses, but were used in the data sets for individual genes when there was no evidence of within-gene recombination. We analysed sequences using the general time-reversible model of nucleotide substitution, with rate variation among sites modelled using a gamma distribution and a proportion of invariable sites (GTR +г_4_+I). This model was selected in R [44] using the Bayesian information criterion, which has been shown to perform well in a variety of scenarios [45]. Branch support was evaluated by bootstrap analysis based on 100 pseudoreplicates.

For the sake of comparison, we analysed a subset of the data using the Bayesian phylogenetic method in BEAST version 1.4.7 [46]. We also did analyses using neighbor-joining in PHYLIP version 3.5c [47].

### Estimation of Substitution Rates and Divergence Times

Substitution rates and divergence times were estimated from various subsets of the sequence data. We analysed individual alignments of the HC-Pro, protein 3 (P3), NIb, and “coherently-evolving” CP (cCP) genes, which included sequences from both TuMV-OM and TuMV-BI isolates. For some analyses, however, we distinguished between TuMV isolates from brassicas and those from non-brassicas. We used the Bayesian phylogenetic method in BEAST to estimate substitution rates and the times to most recent common ancestors (TMRCAs). Data sets were analysed using both strict and relaxed (uncorrelated exponential and uncorrelated lognormal) molecular clocks [48]. We used the software Path-O-Gen version 1.3 (http://tree.bio.ed.ac.uk/software/pathogen/) to test for clocklike evolution using regression of root-to-tip distances on viral sampling times. Small R-squared values were obtained for all alignments, indicating that it was necessary to use relaxed-clock models. We also used Bayes factors to compare five demographic models (constant population size, expansion growth, exponential growth, logistic growth, and the Bayesian skyline plot), which were used as coalescent priors.

Posterior distributions of parameters, including the tree, were estimated by Markov chain Monte Carlo sampling. Samples were drawn every 10,000 steps over a total of 100 million steps, with the first 10% of samples discarded as burn-in. Sufficient sampling from the posterior and convergence to the stationary distribution were checked using Tracer (http://tree.bio.ed.ac.uk/software/tracer/).

To obtain reliable estimates of substitution rates and divergence times from time-stamped data, the range of sampling times needs to have been sufficient for genetic change to have occurred [49]. To test for adequate temporal structure in our data sets, we compared our rate estimates with those from ten date-randomized replicates. Following previous studies [50]–[52], we considered a data set to have sufficient temporal structure when the mean rate estimate from the original data set was not contained in any of the 95% credibility intervals of the rates estimated from the date-randomized replicates.

## Results

### Biological Characteristics of TuMV Isolates

The 74 European, 65 East Asian, and 16 other TuMV isolates examined in this study are listed in Table S1. Most *Brassica* plants, but not *Raphanus sativus*, were systemically infected by most isolates ([3], [6], this study). Thus, they were of the B-infecting host-type (pathotype), although they had minor differences in pathogenicity. Only six European isolates were among this group: Cal1, DEU 4, ITA 2, ITA 7, ITA 8, and PV0104. These had been collected in Italy and Germany and had been isolated from *Abutilon* sp., *Calendula officinalis*, *Cheiranthus cheeri*, *R. raphanistrum*, *R. sativus*, and *Lactuca sativa*. In contrast, approximately 90% of isolates from Europe were B host-type ([4], [6], this study).

Nineteen of 74 isolates collected in Europe were from non-brassicas. Four isolates, which we call the OM isolates or TuMV-OM, came from *Orchis* spp.: OM-N was isolated from *Orchis militaris* and OM-A was isolated from OM-N by single-lesion isolations, whereas ORM and OS were isolated from *Orchis morio* and *Orchis simia* plants, respectively. The fact that OM isolates have only been found in orchids in a single glasshouse collection supports the conclusion [17] that the OM isolates did not come from brassica plants growing near the glasshouse, but came from one or more of the orchids in the collection, all of which were from Central or Mediterranean Europe.

Isolates OM-N and OS did not infect brassica test plants despite repeated testing (Table 1). By contrast, isolate ORM occasionally infected *Brassica rapa* and *B. juncea*, but was only detected by reverse transcription-polymerase chain reaction (RT-PCR) and double antibody sandwich enzyme-linked immunosorbent assay (DAS-ELISA) in the upper two uninoculated leaves of the inoculated plants and not in leaves that emerged subsequently. Hence, as no systemic infection with clear symptoms was produced by OM isolates in standard brassica test plants, it was clear that they were biologically distinct from TuMV-BIs. OM isolates were also inoculated to plants of *Camelina sativa* (‘gold of pleasure’) and *Eruca sativa* (rocket), both of which are West Eurasian brassicas that have been grown, unselected, as crop plants for several thousand years. OM and ORM isolates infected both *C. sativa* and *E. sativa*, but the OS isolate only infected *C. sativa*.

**Table 1:** Host reactions of some isolates of *Turnip mosaic virus* from *Orchis* spp. and other hosts.

Host plant				Symptom^a^			
Family	Species	Common name	Seed origin	OM-N^b^	OS	ORM	Standard brassica isolate^c^
*Amaranthaceae*	*Gomphrena globosa* cv. Bicolor rose	Globe amaranth	France	−/−	(CS/−)	ND	(CS/−)
	*G*. *globosa* cv. Strawberry field	Globe amaranth	UK	−/−	−/−	ND	(CS/−)
*Amaryllidaceae*	*Narcissus tazetta* var. *chinensis* cv. Scarlet Gem	Narcissus	Japan	−/−	−/−	−/−	−/−
	*N. tazetta* var. *chinensis* cv. The Winston Churchill	Narcissus	Japan	−/−	−/−	−/−	−/−
*Asteraceae*	*Lactuca sativa* cv. Emrap 231	Lettuce	USA	−/−	−/−	−/−	−/−
	*L. sativa* cv. Salinas 88	Lettuce	Australia	−/−	−/−	−/−	(LI/M, St, D)
*Brassicaceae*	*Brassica chinensis* cv. Choyo	Qing geng cai	Denmark	−/−	−/−	−/−	NLL/BSS, PSF
	*B. juncea* cv. Hakarashina	Mustard	Italy	−/−	−/−	(LI/M, CS)^d^	NLL/M, LD
	*B. napus* cv. Norin-32 go	Oilseed rape	Japan	−/−	−/−	−/−	NLL/−
	*B. narinosa* cv. Tatsuai	Rosette pakchoi	Australia	−/−	−/−	ND	LI/M
	*B. oleracea var. botrytis* cv. Snow crown	Cauliflower	Australia	−/−	−/−	−/−	(LI/M)
	*B. oleracea var. botrytis* cv. Snow queen	Cauliflower	Chile	−/−	−/−	−/−	(LI/M)
	*B. oleracea var. capitata* cv. Ryozan-2 go	Green cabbage	Japan	−/−	−/−	−/−	(LI/M)
	*B. oleracea var. capitata* cv. Sinsei	Green cabbage	Japan	−/−	−/−	−/−	(LI/M)
	*B. oleracea var. capitata* cv. Yalova-1	White cabbage	Turkey	−/−	ND	ND	(LI/M)
	*B. oleracea var. capitata* cv. Zencibasi	Red cabbage	Turkey	−/−	ND	ND	(LI/M)
	*B. oleracea var. capitata* cv. Mohrenkopt	Red cabbage	Turkey	−/−	ND	ND	(LI/M)
	*B. oleracea* L. var. *acephala* DC.	Kale	USA	−/−	−/−	−/−	(LI/M)
	*B. oleracea var. italica* cv. Challenger	Broccoli	Japan	−/−	−/−	−/−	(LI/M)
	*B. oleracea var. italica* cv. Pixcel	Broccoli	Chile	−/−	−/−	−/−	(LI/M)
	*B. pekinensis* cv. Kyoto-3 go	Chinese cabbage	Japan	−/−	−/−	−/−	(LI/M, Y)
	*B. pekinensis* cv. Nozaki-1 go	Chinese cabbage	Japan	−/−	−/−	−/−	(LI/M, Y)
	*B. pekinensis* cv. unknown	Chinese cabbage	Turkey	−/−	ND	ND	(LI/M, Y)
	*B. rapa* cv. Hakatasuwari	Turnip	Japan	−/−	−/−	(LI/CS)^d^	(LI/M)
	*B. rapa* cv. Ada-202	Turnip	Turkey	−/−	ND	ND	(LI/M)
	*Camelina sativa* cv. Calena	Gold of Pleasure	Austria	LI/VC, M, St	LI/VC,M, St	LI/VC,M, St	LI/VC,M, St
	*Camelina sativa* cv. Suneson	Gold of Pleasure	USA	(LI/VC, M, St)	ND	ND	LI/VC,M, St
	*Cheiranthus cheiri* cv. Vega Yellow	Wallflower	Japan	−/−	ND	−/−	(LI/M)
	*Eruca sativa* cv. Odyssey	Rocket	Italy	(CS, NS/CS, NS, M)	−/−	(LI/M,CS)	(LI/M)
	*E. sativa* cv. Unknown	Rocket	Denmark	CS, NS/CS,M,St	−/−	(LI/mM)	(LI/M)
	*E. sativa* cv. Izmir	Rocket	Turkey	(CS, NS/CS, NS, M)	ND	ND	(LI/M)
	*E. sativa* cv. Balikesir	Rocket	Turkey	(CS, NS/CS, NS, M)	ND	ND	(LI/M)
	*E. sativa* cv. Unknown	Rocket	Turkey	(CS, NS/CS, NS, M)	−/−	ND	(LI/M)
	*E. sativa* cv. unknown (Greek local variety)	Rocket	Greece	−/−	ND	ND	−/−
	*Matthiola incana* cv. Hatsubeni	Stock	Japan	−/−	ND	ND	LI/Mo, M
	*Nasturtium officinale* cv. unknown	Watercress	Denmark	−/−	ND	−/−	(LI/M)
	*Raphanus sativus* cv. Akimasari	Japanese radish	Japan	−/−	−/−	−/−	(LI/M)
	*R. sativus* cv. Everest	Chinese radish	USA	−/−	−/−	−/−	(LI/M)
	*R. sativus* cv. Karagulle	Black radish	Turkey	−/−	ND	ND	(LI/M)
	*R. sativus* cv. Kartopu	White radish	Turkey	−/−	ND	ND	(LI/M)
	*R. sativus* cv. Kuromaru-kun	Black radish	Italy	−/−	ND	ND	(LI/M)
	*R. sativus* cv. Comet	Red small radish	USA	−/−	−/−	−/−	(LI/M)
	*R. sativus* cv. Taibosobutori	Japanese radish	New Zealand	−/−	−/−	−/−	(LI/M)
	*R. sativus* cv. Tenan-koshin	Chinese radish	Italy	−/−	−/−	−/−	(LI/M)
	*Rapistrum rugosum* (L.) All.	Annual bastard cabbage	Iran	−/−	ND	ND	(LI/M)
	*Sisymbrium loeselii* L.	Small tumbleweed-mustard	Iran	−/−	ND	ND	(LI/M)
*Chenopodiaceae*	*Chenopodium quinoa*	Quinoa	Japan	CS/−	CS/−	CS/−	CS/(M)
	*C. amaranticolar*	*Chenopodium album* var. album	Japan	CS/−	CS/−	CS/−	CS/(M)
*Dioscoreaceae*	*Dioscorea japonica*	Japanese yam	Japan	−/−	−/−	−/−	−/−
*Liliaceae*	*Allium fistulosum* cv. White tower	Green onion	Chile	−/−	−/−	−/−	−/−
	*A. fistulosum* cv. Kujyo-futo	Green onion	Chile	−/−	−/−	−/−	−/−
*Orchidaceae*	*Orchis graminifolia*	grass-like leaved orchis	Japan	−/−	−/−	−/−	−/−
*Solanaceae*	*Nicotiana benthamiana*		Japan	LI/M	NS/M	LI/M	LI/M
	*N. clevelandii*		Japan	NR/NR, Mo	LI/M	NR/NR, M	NR/NR, M
	*N. tabacum* cv. Sumsun		Japan	ND	CS/−	CS/−	(CS/−)
	*N. tabacum* cv. Xanthi nc		Japan	−/−	CS/−	CS/−	(CS/−)

a Reaction of inoculated leaves/uninoculated upper leaves. At least three plants were inoculated. BSS; Black stem streak, CS; Chlorotic spot, D; Dead, LD; Leaf deformation, LI; Latent infection, M; Mosaic, Mo; Mottle, ND: Not detected, NLL; Necrotic local lesion, NS; Necrotic spot, NR; Necrotic ringspot, PSF; Pod set failure, St; Stunting, VC; Vein clearing, -; No infection, (); Occasionally. All the leaves were examined for *Turnip mosaic virus* (TuMV) infection by DAS-ELISA. All the absorbance values at 405 nm of DAS-ELISA were greater than 1.0 2.5 hrs after adding substrate.

b OS and ORM isolates are aphid transmissible but OM-N isolate is not.

c Typical symptom for most of TuMV brassica isolates.

d Viruses were detected only in upper two leaves, and not in the third.

Isolates OS and ORM were transmitted by *Myzus persicae* (Sulzer) and *Aphis gossypii* (Glover) in a non-persistent manner, whereas OM-N and OM-A isolates were not, even though they were tested several times using more than 100 apterous aphids for each test; *E. sativa* and *C. sativa* were used as source and test plants. The three OM isolates were also mechanically inoculated to, but failed to infect, leaves of *Narcissus tazetta* var. *chinensis* (narcissus), *Dioscorea japonica* (Japanese yam), and *Allium fistulosum* (green or Welsh onion, scallion), which are the original hosts of NYSV, JYMV, and ScMV, respectively, and form the sister group to all TuMVs.

### Genome Sequences

We sequenced the genomes of 48 TuMV isolates from Europe amd five from USA. We analysed these along with 102 other genomic TuMV sequences, mostly of East Asian isolates [4], obtained from the international sequence databases (Table S1). Most were 9798 nt in length; a few were one to three nucleotides shorter in the terminal UTRs, whereas those of OM isolates were 6 nt (i.e., two codons) shorter in the polyprotein region. All of the motifs reported in potyvirus genes, encoded proteins, and the ‘Pretty Interesting *Potyviridae* ORF’ (P3N-PIPO) [9] were present. The P1 gene and its encoded protein were the most variable, and these had few totally conserved residues or compact motifs. The P3 gene and its encoded protein were only slightly less variable [4], [8]. The sequences are available in the GenBank, EMBL, and DDBJ databases with Accession Codes AB701690-AB701742.

### Phylogenetic Analyses

**Figure 1:**
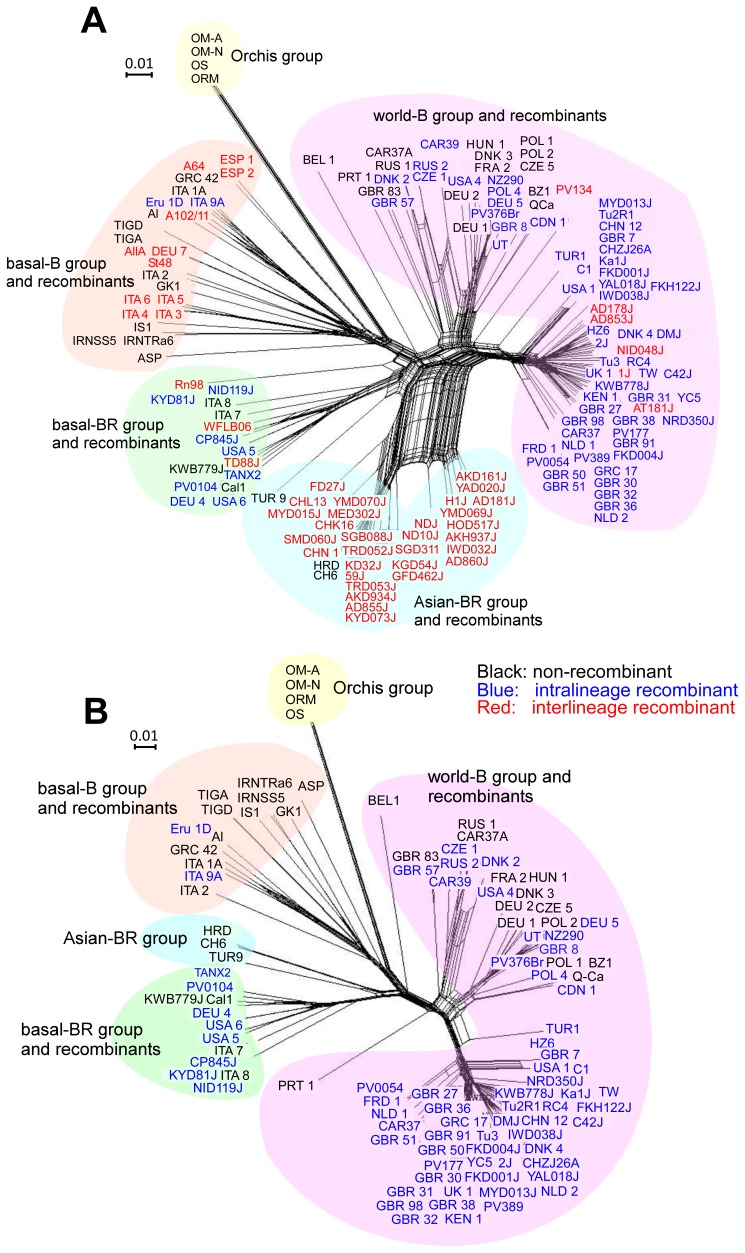
Split-decomposition phylogenetic networks. Networks inferred from (A) polyprotein sequences of 155 *Turnip mosaic virus* (TuMV) isolates and (B) 105 sequences remaining after removing interlineage recombinants. The isolates of non-recombinants (acronyms in black), intrarecombinants (acronyms in blue) and interrecombinants (acronyms in red) are separately listed.

Separate phylogenetic trees were estimated for the polyproteins and for the individual HC-Pro, P3, NIb, and CP genes/regions of the 155 isolates. Inconsistent and poorly supported relationships among the resulting trees indicated that some isolates were recombinants, as found previously [3], [53]. Accordingly, we checked the sequences for recombination using split decomposition [34]. The OM isolates formed a single non-recombinant lineage distinct from all the TuMV-BI lineages, and closest to the basal-B lineage (Figure 1A). The extent of the reticulations at the base of the world-B and Asian-BR lineages suggests that most of these sequences are recombinants.

Further analyses using recombination-detection methods confirmed that many of the sequences were recombinants, with only 37 of the sequences showing no significant evidence of recombination. Fifty sequences were interlineage recombinants (i.e., had ‘parents’ from different lineages; red names in Figure 1A), whereas 68 sequences were intralineage recombinants (i.e., had ‘parents’ from the same lineage; blue names in Figure 1A). When the interlineage recombinant sequences were removed, and the remaining sequences analysed again by split decomposition, the branching patterns of the major lineages were resolved (Figure 1B).

**Figure 2:**
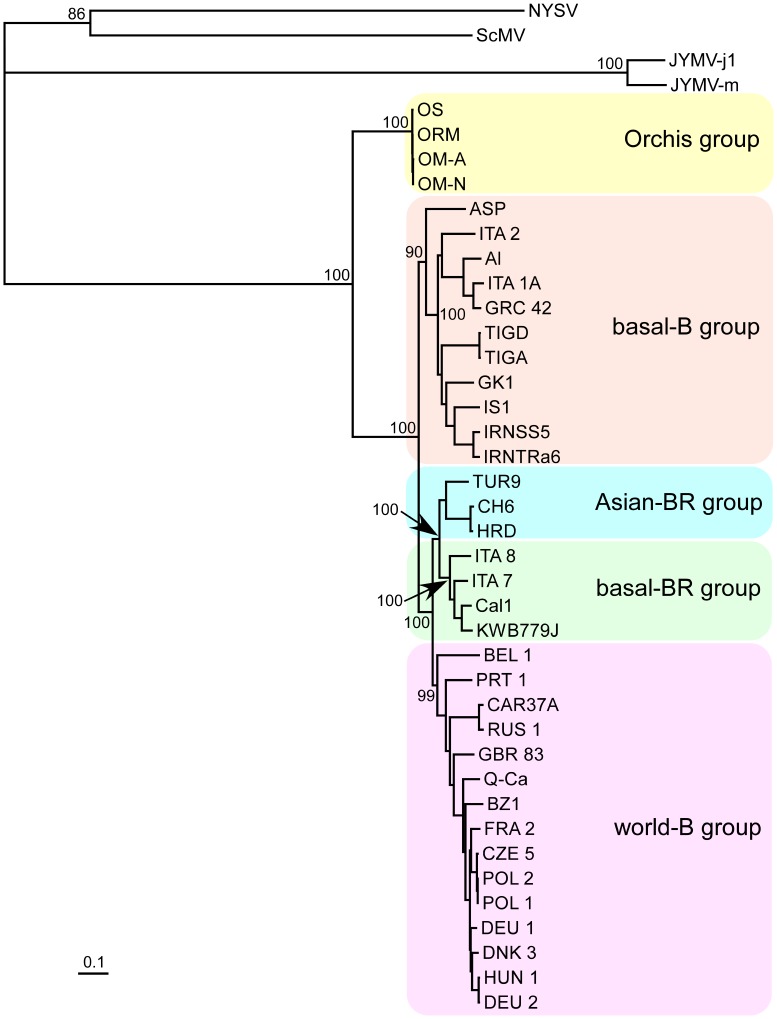
Maximum-likelihood tree of the complete polyprotein sequences of 37 non-recombinant *Turnip mosaic virus* isolates. Nodes are labelled with bootstrap support percentages.

Figure 2 shows a maximum-likelihood tree of the amino acid sequences encoded by the few genomic sequences that had no evidence of recombination. It confirms that the OM isolates form a monophyletic lineage that is sister to the TuMV-BI lineages and closest to the basal-B group.

No recombination sites were detected in the 5′ and 3′ non-coding regions. However, they were found throughout the coding regions of many of the genomes (Table S2), especially in the P1 gene and CI-VPg regions as reported previously [4]. Therefore, the HC-Pro, P3, NIb, and CP genes/regions of the genomes that were not intralineage recombinants, and showed no intragenic recombination, were selected for phylogenetic analyses using maximum likelihood in PhyML version 3 [43] and neighbor-joining in PHYLIP [47]. The resulting trees grouped the TuMV-BI sequences into the four major groups previously reported [3], [4], with the OM isolates grouped as a monophyletic sister lineage to all of the other TuMV lineages. An exception to this occurred in the HC-Pro trees, where the OM lineage was sister to the basal-B lineage. All of these topologies were supported by high bootstrap values.

These results raised the question as to whether the OM isolates were closer to the TuMV-BIs or to the outgroup viruses. In a maximum-likelihood phylogeny of 37 non-recombinant TuMV-BI genomes, the four TuMV-OM genomes, and the four outgroup genomes, the mean patristic distances between the outgroup sequences and the BI and OM genomes were 7.97±1.06 and 7.63±1.09 subs/site, respectively, but only 1.01±0.03 subs/site between the BI and OM genomes. Thus, the BI and OM genomes represent distinct populations that are much closer to one another than to the outgroup viruses. Figure S1 shows in detail that the sequences of the OM isolates are closer to those of TuMV isolates throughout the genome, and both are very different from the outgroup genomes. They are also closer in terms of other characteristics, such as the lengths of the genomes and each gene, especially those of the P1 and CP genes (Table S3). In addition, the protein cleavage sites of the OM and TuMV-BI isolates are more similar to each other than to those of outgroups (Table S4). Thus, we conclude that OM isolates are close to, although biologically distinct from, the TuMV-BIs.

Several of the BI isolates were isolated from non-brassica hosts other than orchids, but like other TuMV-BIs, they infected most brassicas. Most had been collected in Europe and most were from the basal-B lineage. However, a Monte Carlo ‘provenance randomization’ test [16], [54] showed that they did not significantly cluster in maximum-likelihood trees.

### Evolutionary Rates and Timescales

We used a Bayesian phylogenetic approach to estimate TMRCAs and nucleotide substitution rates of the individual genes of the TuMV-BI and TuMV-OM isolates. For all four data sets, a relaxed clock model was found to fit the data better than the strict clock model (Table S5). Notably, for all four data sets, similar posterior means were obtained with all demographic models and for both uncorrelated lognormal and uncorrelated exponential clock models. The best-supported demographic model for the HC-Pro, P3, and NIb TuMV sequences was that of constant population size, whereas for the cCP region [16], [55], [56] a model of exponential growth received the strongest support (Tables 2 and S5).

**Table 2:** Details of the data sets used for estimation of nucleotide substitution rate and time to the most recent common ancestor.

	Region^a^				
Parameter	HC-Pro	P3	Nib	cCP	cCP+16
Sequence length (nt)	1374	1065	1551	711	759
No. of sequences	108	109	115	113	113
Sampling date range	1968–2007	1968–2007	1968–2007	1968–2007	1968–2007
Best-fit substitution model	GTR+I+г	GTR+I+г	GTR+I+г	GTR+I+г	GTR+I+г
Best-fit molecular clock model	Relaxed Uncorrelated Exponential	Relaxed Uncorrelated Exponential	Relaxed Uncorrelated Exponential	Relaxed Uncorrelated Exponential	Relaxed Uncorrelated Exponential
Best-fit population growth model	Constant Size	Constant Size	Constant Size	Exponential Growth	Expansion Growth

a HC-Pro; Helper component-proteinase protein. P3; Protein 3. NIb; Nuclear inclusion b protein. cCP; Coherently-evolving coat protein. cCP+16; Sequences that include the 16 codons (48 nucleotides) at 5′-terminus immediately adjacent to cCP.

Preliminary analyses of all 108–115 sequences in each dataset revealed large differences (up to five-fold) in the mean TMRCAs estimated for different genes (Table 3; mixed hosts), despite care having been taken to remove recombinant sequences. Thus, these results were unable to provide a reliable estimate of the emergence time of TuMV, which was the principal objective of this project. The results were no more consistent when the analyses were confined to genes from the 28 complete genomic sequences that had collection date and no significant recombination signals (data not shown). We found that different regions of the CP gene sequences gave quite different TMRCA estimates: the cCP region [16], [56] yielded much smaller TMRCAs than sequences that included the 16 codons (48 nts) immediately adjacent to the cCP region (Table 3; cCP+16 results).

**Table 3:** Estimates of the substitution rates and times to the most recent common ancestor (TMRCA) for TuMVs isolated from various hosts, from only *Brassicaceae* or from non-*Brassicaceae*, or from orchids and *Brassicaceae*.

			Nucleotide substitution rate^a^		TMRCA (years)	
TuMV isolates from:	Region^b^	No. of sequences	mean	95% CI^c^	mean	95% CI
a mixture of hosts	HC-Pro	108	1.07×10^−3^	5.82×10^−4^–1.56×10^−3^	819	258–1643
	P3	109	1.08×10^−3^	5.59×10^−4^–1.60×10^−3^	1071	279–2511
	NIb	115	7.04×10^−4^	3.73×10^−4^–1.00×10^−3^	1330	342–2920
	cCP	113	6.12×10^−4^	3.41×10^−4^–8.93×10^−4^	271	127–470
	cCP+16	113	1.14×10^−4^	4.98×10^−6^–2.50×10^−4^	4070	335–11643
*Brassicaceae* only	HC-Pro	88	1.14×10^−3^	4.49×10^−4^–1.75×10^−3^	800	192–1775
	P3	88	1.17×10^−3^	4.97×10^−4^–1.89×10^−3^	829	196–1815
	NIb	93	7.61×10^−4^	3.26×10^−4^–1.18×10^−3^	928	191–1936
	cCP	91	5.63×10^−4^	1.57×10^−4^–9.57×10^−4^	446	110–973
	cCP+16	92	9.91×10^−5^	1.84×10^−6^–2.59×10^−4^	4027	157–12650
non-*Brassicaceae*	HC-Pro	20	1.97×10^−3^	9.91×10^−6^–4.31×10^−3^	765	63–1855
	P3	21	2.71×10^−3^	3.81×10^−6^–6.19×10^−3^	959	56–1967
	NIb	22	1.19×10^−3^	2.65×10^−7^–3.16×10^−3^	3485	66–6997
	cCP	22	1.61×10^−3^	4.44×10^−4^–2.90×10^−3^	186	49–414
	cCP+16	21	1.65×10^−3^	3.76×10^−4^–3.02×10^−3^	178	46–405
Orchids and *Brassicaceae*	HC-Pro	92	1.13×10^−3^	6.31×10^−4^–1.68×10^−3^	754	223–1555
	P3	92	1.09×10^−3^	4.93×10^−4^–1.76×10^−3^	1030	241–2219
	NIb	97	8.63×10^−4^	4.44×10^−4^–1.28×10^−3^	1025	240–2365
	cCP	95	6.85×10^−4^	3.85×10^−4^–1.02×10^−3^	284	114–533
	cCP+16	95	2.47×10^−4^	4.66×10^−6^–6.47×10^−4^	3041	170–10631

a substitutions/site/year.

b HC-Pro; Helper component-proteinase protein. P3; Protein 3. NIb; Nuclear inclusion b protein. cCP; Coherently-evolving coat protein. cCP+16; Sequences that include the 16 codons (48 nucleotides) at 5′-terminus immediately adjacent to cCP.

c 95% credibility interval.

To investigate whether there is a temporal signal in the data sets, we used two available methods. First, we calculated the correlation between root-to-tip distances and sampling date using Path-O-Gen. Second, we analysed replicate data sets in which the ages of the sequences were randomized, as done in a number of recent studies [50]–[52]. Parameter estimates from each dataset were only considered reliable if the mean posterior rate obtained using the original sample dates was outside the 95% credibility intervals of rates estimated from the date-randomized data. The HC-Pro, P3, NIb, and cCP, but not the cCP+16, data sets passed this date-randomization test. In addition, the rate estimates from the original data sets had much smaller 95% credibility intervals than those from the date-randomized data sets (Figure S2). The mean estimated substitution rates for the three largest genes of all TuMVs, excluding the results for the non-brassica isolates, are HC-Pro 1.11×10^−3^, P3 1.11×10^−3^, and NIb 0.78×10^−3^ subs/site/year (Table 3).

Analysis of sequences of TuMV-BI isolates from non-brassicas gave more variable results (Table 3; mean TMRCA estimates cCP 186 years to NIb 3485 years) than those from the isolates from brassicas (Table 3; mean TMRCA estimates cCP 446 years to NIb 928 years). This might be due to sampling variability, however, because there were only around 21 isolates in each non-brassica dataset compared with around 90 isolates in brassica datasets. We checked this by estimating the TMRCAs of 10 subsets of 21 sequences randomly selected from the 88 P3 genes from brassica isolates. These subsets produced widely variable mean estimates of TMRCAs (Figure S3). Most of these estimates are lower than that from the complete 88 sequence set, but two were considerably higher. For example, mean TMRCA estimates ranged from 99 years to 10,531 years (mean, 1385 years; logarithmic mean 354 years), compared with a mean estimate of 829 years for the complete set of 88 P3 sequences. Our analyses showed that the estimated TMRCAs and evolutionary rates for the three largest genes of each data set (i.e., HC-Pro, P3, and NIb) were always more similar to each other than to those from the cCP gene. We suggest that the temporal signal in the cCP sequences is less reliable because the cCP gene is around half the length of the others and has the lowest evolutionary rate (Table 3). Accordingly, we excluded the estimates obtained from this gene when inferring the time of divergence of the TuMV-BI and TuMV-OM lineages.

The three largest genes provided two estimates of the divergence time of the TuMV-BIs and TuMV-OMs. The sequences from the “mixture of hosts” isolates (Table 3) gave a mean date estimate of 1073 YBP. The sequences from the “brassicas and orchids, but not non-brassicas” (Table 3), namely all isolates except those from non-*Brassicaceae*, gave a mean date estimate of 936 YBP. Even though the latter data set comprised a smaller number of sequences, the estimates differed less among genes. Therefore, in the absence of a clear reason for distinguishing between these two sets of results, we take the mean of these two values, 1005 YBP (mean 95% credibility interval from 264 YBP to 2203 YBP), as the most likely date of emergence of TuMV-BI. In addition, the “brassicas only” data set gives, for the three largest genes, a consistent estimate of the divergence time of the main lineages of TuMV-BIs of about 852 YBP (mean 95% CI from 193 YBP to 1842 YBP; mean TMRCAs of 800 YBP for HC-Pro, 829 YBP for P3, and 928 YBP for NIb).

## Discussion

Our phylogenetic analyses have shown that a group of isolates from European orchids form a small monophyletic sister group to all the TuMV isolates that readily infect brassicas. The same phylogeny was inferred by all analytical methods (maximum likelihood, neighbor-joining, and Bayesian inference), except for some analyses of the HC-Pro data, and was also inferred with the full genomes, the encoded polyproteins, or individual genes. The TuMV-OMs and TuMV-BIs form phylogenetically distinct sister lineages within the TuMV group of potyviruses, which also includes the more distantly related JYMV, NYSV, and ScMV.

The TuMV-OM isolates came from orchids grown for two years in a glasshouse collection of geophyte orchids at Celle, Germany. It is uncertain whether one or more of these species are hosts of OM viruses in nature because the same virus was also found in other orchid species in the same collection. Nonetheless, all of the infected plants have overlapping natural distributions in central and southern Europe within the region identified as the likely ‘centre of emergence’ of TuMV-BIs.

Earlier detailed serological tests had shown that TuMV-OM was most closely related to, but distinct from, TuMV [17]. This was supported by sequence analysis of the coat protein gene of the OM isolate [18]. In the present study, we have confirmed and extended these earlier results and found that the TuMV-OM isolates are biologically distinct from all TuMV-BI isolates. Only one orchid isolate infected brassicas systemically, but did not produce symptoms, and none infected the monocotyledonous hosts of the outgroup viruses, JYMV, NYSV, and ScMV. Hence, the TuMV-OMs are biologically and phylogenetically distinct from TuMV-BIs, and should perhaps be considered a separate viral species (http://ictvonline.org/codeOfVirusClassification_2002.asp).

The immediate ancestor of the sister lineages was probably either brassica-infecting and spread to orchids, or orchid-infecting and spread to brassicas. In the absence of direct evidence, we conclude that the latter scenario is more likely because it involves fewer host changes. Also the TuMV-OM population has the characteristics one would expect of the source of the TuMV-BI lineage: it is phylogenetically distinct but closely related; it is found in a host more closely related to those of known outgroup viruses than to TuMV-BI, although some isolates can infect species of *Brassicaceae*; and it is from western Eurasia, matching the likely location of the emergent virus population.

When attempting to estimate the timing of the divergence between TuMV-BI and TuMV-OM lineages, our initial Bayesian results were inconsistent but instructive. We found sufficient temporal structure among samples from the genome region encoding the coherently-evolving C-terminal part of the CP, but this data set still gave TMRCA estimates that differed substantially from the other genes. This was possibly due to the small size of the data set, in terms of both sequence length and sample size. Some of the variation in rates among genes might be due to differences in sites under purifying selection, which appears to have had a strong effect on the four genes. However, estimates of evolutionary rates were very similar among the three largest genes. The effects of purifying selection, whereby younger branches of the tree tend to carry an elevated number of transient polymorphisms, might have led to an overestimation of the mutation rate in our analysis [57], [58]. However, our rate estimates are very close to the mean rate found in a survey of virus studies [59], but higher than those reported earlier for *Zucchini yellow mosaic virus* (ZYMV) (5.0×10^−4^ subs/site/year) and for a group of potyviruses (1.15×10^−4^ subs/site/year) [16], [54].

The OM and BI lineages were estimated to have diverged around 1005 YBP; long before TuMV was first isolated in North America in 1921 [60]. The phylogenetic trees (Figures 1 and 2) indicate that the TuMV-BIs subsequently radiated around 850 YBP to give the four present-day lineages. The basal-B lineage, isolates of which have been found most often in Europe, radiated soon after the TuMV-BIs lineages were established. The other three lineages diversified more recently to be found in other parts of the world.

Although the TuMV population of the world has been more thoroughly sampled than that of any other plant virus, it is important to realize that only four TuMV-OM isolates have been examined so far. Accordingly, our conclusions about its role in the evolution of TuMV must be treated with caution. It is difficult to obtain independent evidence to corroborate our estimate of the time of divergence of TuMV and TuMV-OM. Nevertheless, the conclusion that TuMV-BI emerged in Western Europe around 1000 YBP is congruent with historical records of conditions existing at that time. Before then agriculture was small-scale and the landscape was dominated by natural ecosystems. Eurasian agriculture had first developed around 11,000–10,500 YBP in the region bordering the eastern Mediterranean from Turkey to Egypt (i.e., the Levant), and was based on the domestication of cereals, legumes, flax and grazing animals. It dispersed north and west into Eurasia around 8,500 YBP and finally to the north and west fringes of Europe around 6,000 YBP [61]–[63].

The genetically complex group of brassica hybrids now grown as crops, including turnips, was domesticated from about 4000 YBP, probably around the Mediterranean and cooler parts of northwest Europe [64]–[68], and became a staple of the diet of humans and domesticated animals. In the Medieval Warm Period, 1050–750 YBP, there was a warming of the climate in West Eurasia and a great increase in both the human population and the extent of agriculture; forests and marshes were cleared and cultivated [63]. This corresponds to the period during which, we suggest, the first TuMV-BI emerged. The landscape changed from isolated farming settlements set in woodlands to a landscape of contiguous farmlands with minor dispersed woodlands, and this would have fostered the spread of crop diseases. The conditions would have been ideal for a potyvirus like TuMV-OM, able to infect brassicas, to emerge from wild hosts and adapt to the increased population of brassicas provided by crops and their weeds.

Furthermore, potyviruses are spread by aphids as they non-specifically probe plants seeking suitable hosts on which to breed. Most potyviruses are spread by aphids from Aphidineae, a group that is unusual among phytophagous insects in that most species alternate between woody winter hosts and herbaceous summer hosts, and thus aphidines were also fostered by the conditions of early broadscale agriculture [16]. The spread of TuMV-BIs to produce the present global distribution of the virus probably had to wait until international maritime trade was established after the discovery of the Americas and routes to the Far East by European adventurers around 500 YBP. Similar analyses of the relationships and evolutionary timescales of the bean common mosaic and potato virus Y groups of potyviruses also concluded that most species had arisen in the last 1000 to 3000 years, and had involved similarly unknown host:virus:landscape specificities [8], [69].

Further testing of wild populations of European orchids and brassicas will enable us to determine which species are the primary host or hosts of TuMV-OM, and also whether any intermediates in the adaptation of this virus to brassicas have survived. Furthermore, because the primary hosts of all potyviruses are monocotyledonous bulb and grass species from Western Eurasia, a broad survey of such plants might reveal other relictual potyvirus populations and provide insight into the intermediate stages of potyvirus evolution.

## Supporting Information

Figure S1 **Similarity plot with OM-N genome sequence as the query isolate.** Graph of the similarities between the genome sequence of OM and those of Al (red) and UK1 (blue) isolates, and *Japanese yam mosaic virus* (JYMV) (light green), *Scallion mosaic virus* (ScMV) (pink), and *Narcissus yellow stripe virus* (NYSV) (dark green). The similarities were estimated using SIMPLOT 3.5.1 with a window size of 200 nt.
(TIF)

Figure S2 **Estimates of nucleotide substitution rates.** Mean estimates and 95% credibility intervals are shown. These were estimated from 108 helper component proteinase (HC-Pro) genes, 109 protein 3 (P3) genes, 115 nuclear inclusion b protein (NIb) genes, 113 coherently-evolving CP (cCP) genes, and 113 cCP+16 genes (see text) from non-recombinant and dated gene sequences of isolates obtained from species of non-brassicas and brassicas. In each set of estimates, the first is based on the original data, whereas the remaining ten values are from date-randomized replicates. The 95% credibility intervals of the estimates from the date-randomized replicates do not overlap with the mean posterior estimate from the original data set. In addition, the lower tails of the credibility intervals are long and tend towards zero. These features suggest that there is sufficient temporal structure in the original data sets for rate estimation.
(TIF)

Figure S3 **Estimated times to the most recent common ancestors of 88 P3 gene sequences and randomly selected subsets of 21 sequences.** The leftmost data point shows the estimate from the original 88 P3 sequences. The remainining 10 data points show the estimates for each of 10 randomly selected sets, each comprising 21 sequences. Error bars indicate 95% credibility intervals.
(TIF)

Table S1 ***Turnip mosaic virus*** **isolates analysed in this study.**
(DOC)

Table S2 **Recombination sites in full genomic sequences.**
(DOC)

Table S3 **Comparisons of the lengths of the genes of Japanese yam mosaic virus (JYMV), Narcissus yellow stripe virus (NYSV), Scallion mosaic virus (ScMV) and Turnip mosaic virus (TuMV).**
(DOC)

Table S4 **Comparisons of the amino acids at the polyprotein cleavage sites of** ***Japanese yam mosaic virus*** **(JYMV),** ***Narcissus yellow stripe virus*** **(NYSV),** ***Scallion mosaic virus*** **(ScMV) and** ***Turnip mosaic virus*** **(TuMV).**
(DOC)

Table S5 **Detailed results of the Bayesian coalescent analysis.**
(DOC)
