# Supplementary figures and images for: Turnip Mosaic Potyvirus Probably First Spread to Eurasian Brassica Crops from Wild Orchids about 1000 Years Ago
Source: PLoS One. 2013 Feb 6;8(2):e55336. doi: 10.1371/journal.pone.0055336 (PMC3566190; doi:10.1371/journal.pone.0055336)

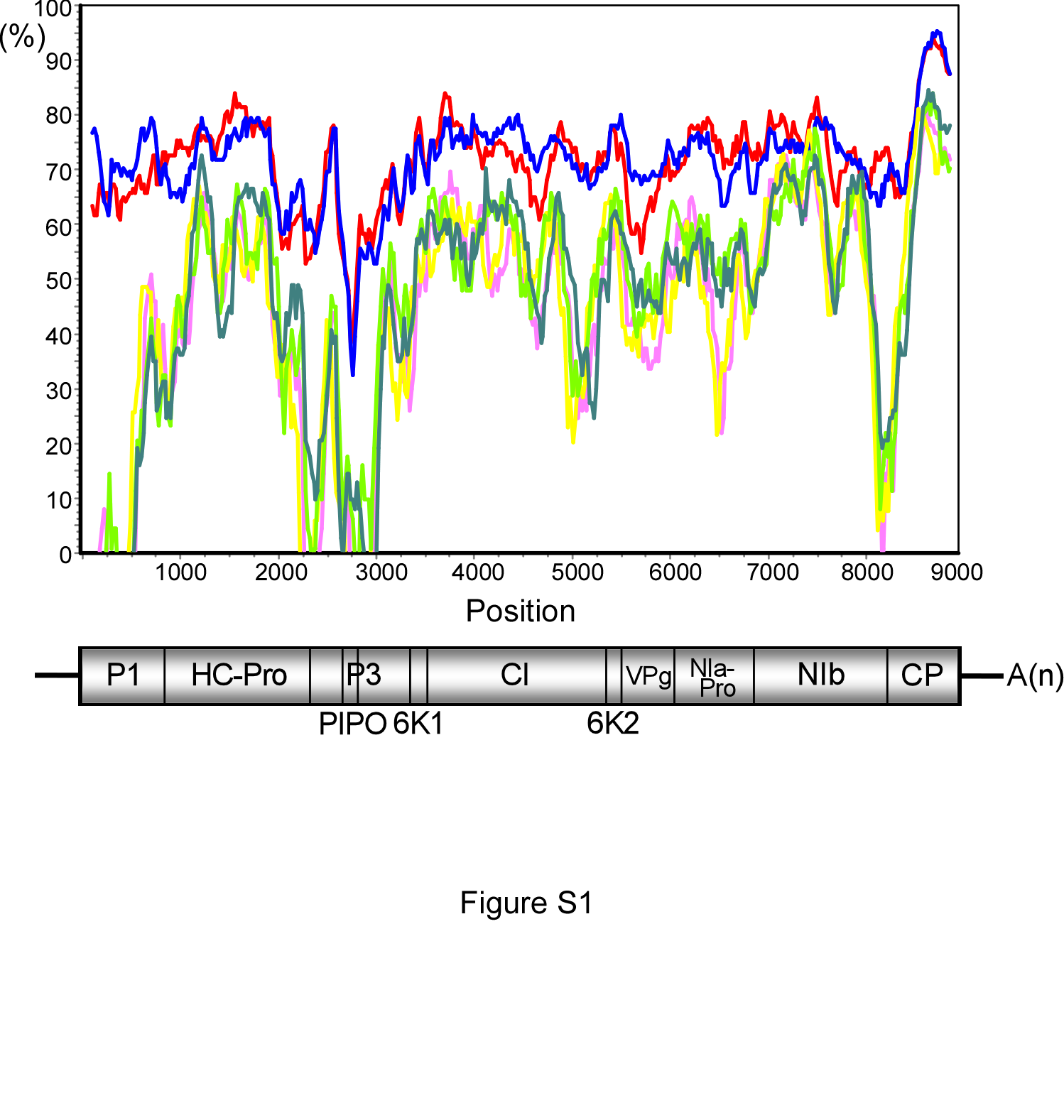

Supplement: Figure S1 — Similarity plot with OM-N genome sequence as the query isolate. Graph of the similarities between the genome sequence of OM and those of Al (red) and UK1 (blue) isolates, and Japanese yam mosaic virus (JYMV) (light green), Scallion mosaic virus (ScMV) (pink), and Narcissus yellow stripe virus (NYSV) (dark green). The similarities were estimated using SIMPLOT 3.5.1 with a window size of 200 nt. (TIF) [file pone.0055336.s001.tif]

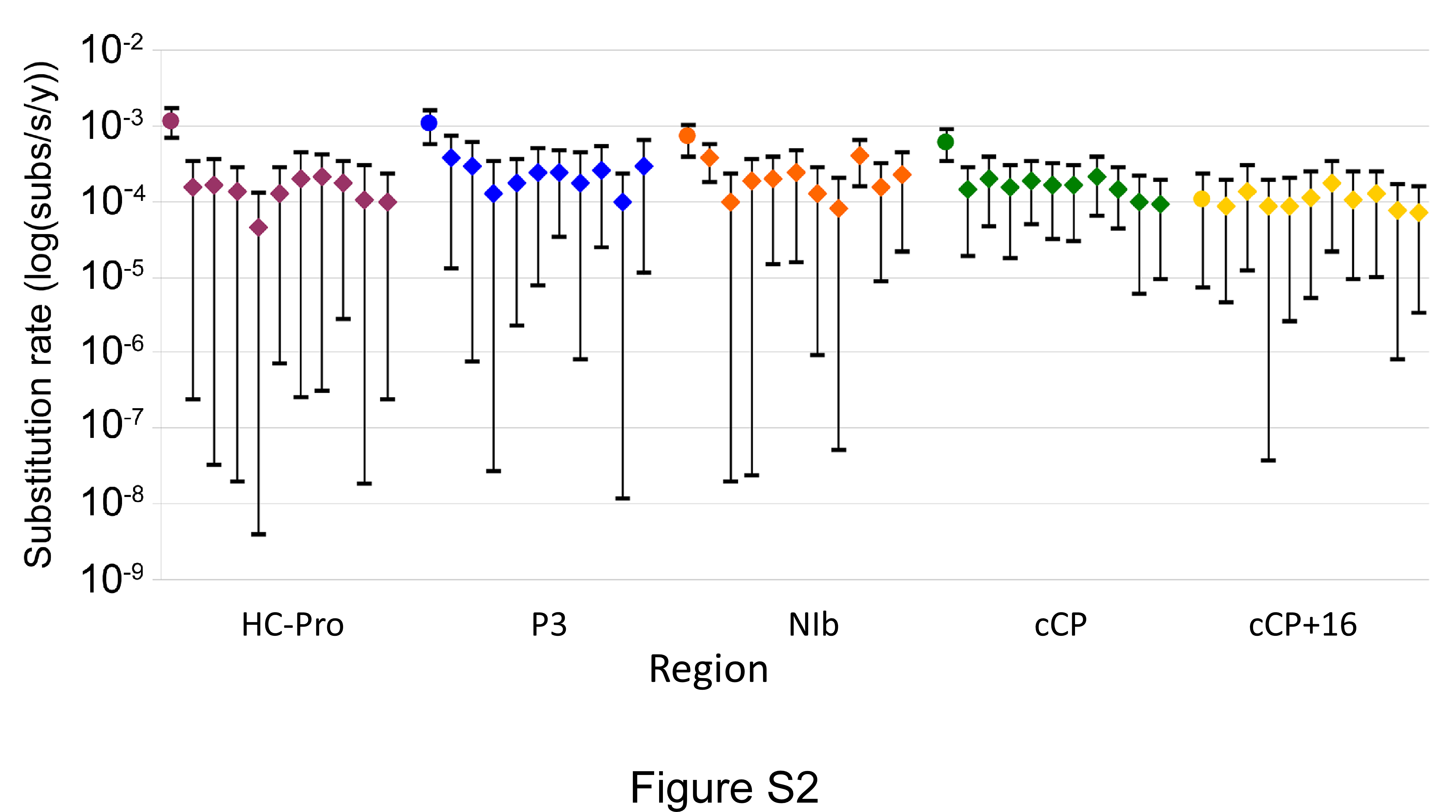

Supplement: Figure S2 — Estimates of nucleotide substitution rates. Mean estimates and 95% credibility intervals are shown. These were estimated from 108 helper component proteinase (HC-Pro) genes, 109 protein 3 (P3) genes, 115 nuclear inclusion b protein (NIb) genes, 113 coherently-evolving CP (cCP) genes, and 113 cCP+16 genes (see text) from non-recombinant and dated gene sequences of isolates obtained from species of non-brassicas and brassicas. In each set of estimates, the first is based on the original data, whereas the remaining ten values are from date-randomized replicates. The 95% credibility intervals of the estimates from the date-randomized replicates do not overlap with the mean posterior estimate from the original data set. In addition, the lower tails of the credibility intervals are long and tend towards zero. These features suggest that there is sufficient temporal structure in the original data sets for rate estimation. (TIF) [file pone.0055336.s002.tif]

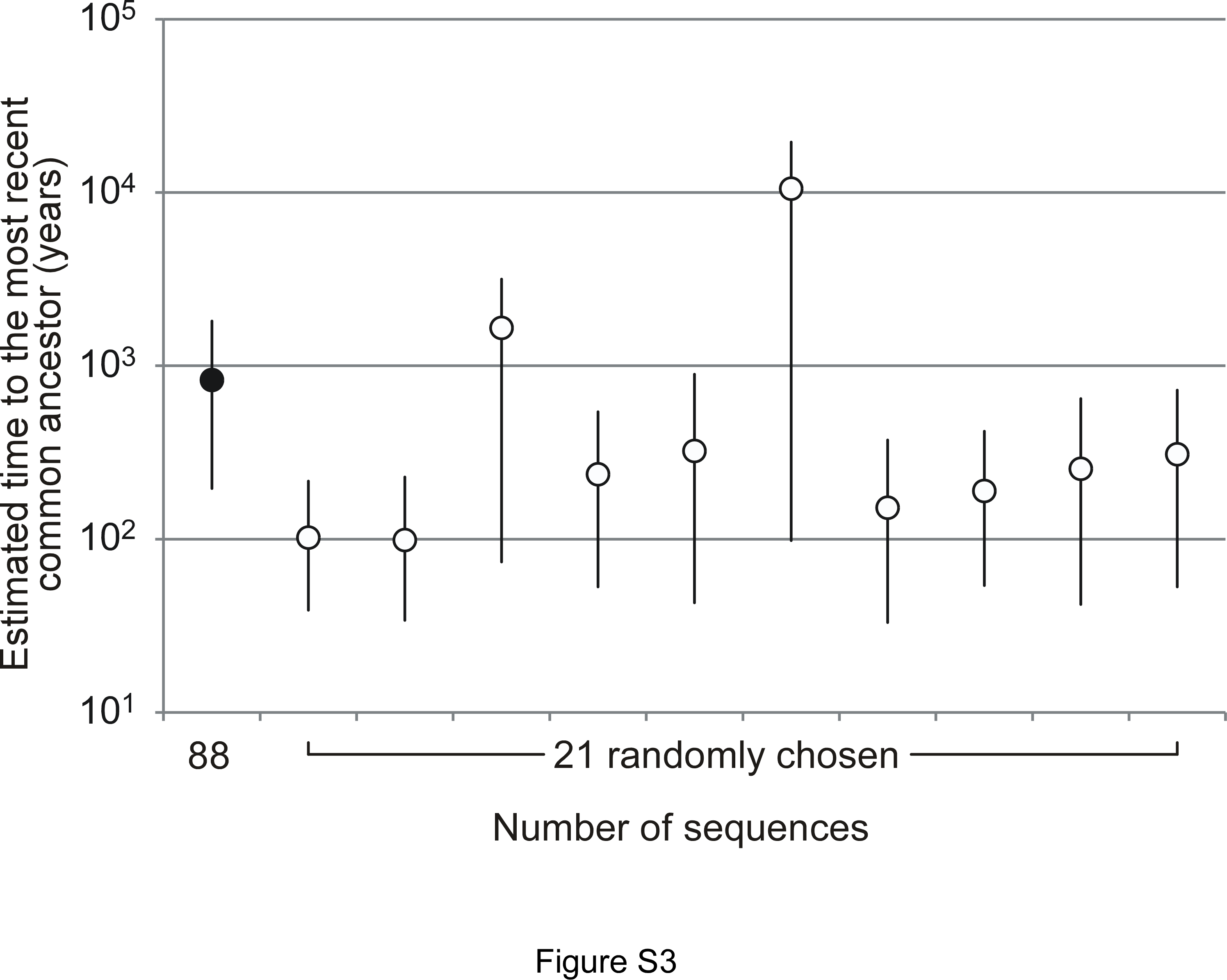

Supplement: Figure S3 — Estimated times to the most recent common ancestors of 88 P3 gene sequences and randomly selected subsets of 21 sequences. The leftmost data point shows the estimate from the original 88 P3 sequences. The remainining 10 data points show the estimates for each of 10 randomly selected sets, each comprising 21 sequences. Error bars indicate 95% credibility intervals. (TIF) [file pone.0055336.s003.tif]
