# Supplementary material for: Turnip Mosaic Potyvirus Probably First Spread to Eurasian Brassica Crops from Wild Orchids about 1000 Years Ago
Source: PLoS One. 2013 Feb 6;8(2):e55336. doi: 10.1371/journal.pone.0055336 (PMC3566190; doi:10.1371/journal.pone.0055336)
Supplement: Table S1 — Turnip mosaic virus isolates analysed in this study. (DOC) [file pone.0055336.s004.doc]

**Table S1.** *Turnip mosaic virus* isolates analysed in this study.

| Isolate | Original host | Location (City, District, Country) | Year of collection | Host type^a^ | Reference^b^ | Accession code |
| --- | --- | --- | --- | --- | --- | --- |
| Europe |  |  |  |  |  |  |
| A102/11 | *Anemone coronaria* | -, Liguria, Italy | 1993 | B | [6] | AB093597 |
| A64 | *A. coronaria* | -, Liguria, Italy | 1991 | B | [6] | AB093599 |
| Al | *Alliaria officinalis* | -, Piedmont, Italy | 1968 | (B) | [6] | AB093598 |
| AllA | *A. officinalis* | -, -, Denmark | 1991 | B |  | this study, AB701694 |
| ASP | *Allium* sp. | Gatersleben, -, Germany | 1995 | B |  | this study, AB701697 |
| BEL 1 | *Rorippa nasturtium-aquaticum* | Wavre, -, Belgium | 1986 | B |  | this study, AB701698 |
| Cal1 | *Calendula officinalis* | -, Liguria, Italy | 1979 | BR | [6] | AB093601 |
| CAR37 | *Cochlearia armoracia* | -, -, Poland | 2004 | Not known | [26] | DQ648592 |
| CAR37A | *C. armoracia* | -, -, Poland | 2004 | Not known | [26] | DQ648591 |
| CAR39 | *C. armoracia* | -, -, Poland | 2004 | Not known |  | EF374098 |
| CZE 1 | *Brassica oleracea* | Ruzyne, -, Czech Republic | 1981 | B | [6] | AB093608 |
| CZE 5 | *B. rapa* | Ceske Budejovice, -, Czech Republic | 1993 | B | [4] | AB252107 |
| DEU 1 | Not known | -, -, Germany | <1976 | B |  | this study, AB701699 |
| DEU 2 | *Raphanus sativus* | -, -, Germany | <1993 | B |  | this study, AB701700 |
| DEU 4 | *Lactuca sativa* | Stuttgart, -, Germany | 1986 | BR |  | this study, AB701701 |
| DEU 5 | *L. sativa* | Monchengladbach, -, Germany | 1991 | B |  | this study, AB701702 |
| DEU 7 | *L. sativa* | Frankfurt,-, Germany | 1994 | B |  | this study, AB701695 |
| DNK 2 | *B. napus* | -, -, Denmark | <1993 | B | [4] | AB252108 |
| DNK 3 | *B. rapa* | -, -, Denmark | 1978 | B |  | this study, AB701703 |
| DNK 4 | *B. rapa* | -, -, Denmark | 1986 | B |  | this study, AB701704 |
| Eru 1D | *Eruca sativa* | Piedmont, Italy | 1991 | B |  | this study, AB701705 |
| ESP 1 | *Eruca vesicaria* subsp. sativa | -, -, Spain | 2001 | B |  | this study, AB701706 |
| ESP 2 | *Sisymbrium orientale* | Las Matas, -, Spain | 2001 | B |  | this study, AB701707 |
| FRA 2 | *B. napus* | -, -, France | <1994 | B |  | this study, AB701708 |
| FRD 1 | *B. oleracea* | -, -, Germany | 1987 | B | [4] | AB252112 |
| GBR 7 | *Rheum rhabarbarum* | Gloucestershire, -, UK | 15.9.1993 | B |  | this study, AB701709 |
| GBR 8 | *Lunaria annua* | Essex, -, UK | 20.4.1994 | B |  | this study, AB701710 |
| GBR 27 | *B. oleracea* | Kimmeridge, Dorset, UK | 24.3.1999 | B |  | this study, AB701711 |
| GBR 30 | *B. oleracea* | Kimmeridge, Dorset, UK | 26.4.1999 | B |  | this study, AB701712 |
| GBR 31 | *B. oleracea* | Chapman’s Pool, Dorset, UK | 26.4.1999 | B |  | this study, AB701713 |
| GBR 32 | *B. oleracea* | Chapman’s Pool, Dorset, UK | 26.4.1999 | B |  | this study, AB701714 |
| GBR 36 | *B. oleracea* | Winspit, Dorset, UK | 18.6.1999 | B | [4] | AB252113 |
| GBR 38 | *B. oleracea* | Winspit, Dorset, UK | 15.7.1999 | B |  | this study, AB701715 |
| GBR 50 | wild *B. oleracea* | Staithes, Yorkshire, UK | 28.9.1999 | B | [4] | AB252114 |
| GBR 51 | wild *B. oleracea* | Staithes, Yorkshire, UK | 28.9.1999 | B |  | this study, AB701742 |
| GBR 57 | wild *B. oleracea* | Llandudno, Conwy, UK | 12.9.2000 | B |  | this study, AB701716 |
| GBR 83 | wild *B. oleracea* | Llandudno, Conwy, UK | 20.8.2002 | B |  | this study, AB701717 |
| GBR 91 | wild *B. oleracea* | Llandudno, Conwy, UK | 20.8.2002 | B |  | this study, AB701718 |
| GBR 98 | wild *B. oleracea* | Winspit, Dorset, UK | 28.8.2002 | B | [27] | EU861593 |
| GK1 | *Matthiola incana* | -, -, Greece | <1989 | B |  | this study, AB701696 |
| GRC 17 | *B. oleracea* | Volos, -, Greece | 1993 | B | [4] | AB252116 |
| GRC 42 | wild *Allium* sp. | -, -, Greece | 1999 | B | [4] | AB252117 |
| HUN 1 | *Alliaria petiolata* | -, -, Hungary | <1996 | B |  | this study, AB701719 |
| ITA 1A | *Brassica ruvo* | -, Campania, Italy | 1990 | B |  | this study, AB701720 |
| ITA 2 | *Cheiranthus cheiri* | -, Campania, Italy | 1992 | BR |  | this study, AB701721 |
| ITA 3 | *B. ruvo* | -, Campania, Italy | 1990 | B | [4] | AB252122 |
| ITA 4 | *B. rapa* | -, Campania, Italy | 1990 | B |  | this study, AB701722 |
| ITA 5 | *B. ruvo* | -, Campania, Italy | 1990 | B |  | this study, AB701723 |
| ITA 6 | *M. incana* | -, Campania, Italy | 1992 | B |  | this study, AB701724 |
| ITA 7 | *R. raphanistrum* | -, Campania, Italy | 1990 | BR | [6] | AB093600 |
| ITA 8 | *Abutilon* sp. | Piedmont, Italy | 09.1993 | BR |  | this study, AB701725 |
| ITA 9A | *Cucurbita pepo* | -, -, Italy | <1995 | B |  | this study, AB701726 |
| NLD 1 | *B. oleracea* | -, -, The Netherlands | <1995 | B | [4] | AB252133 |
| NLD 2 | *B. oleracea* | -, -, The Netherlands | <1995 | B |  | this study, AB701727 |
| OM-A | *Orchis militaris* | Celle, -, Germany | 1981 | DI^c^ |  | this study, AB701691 |
| OM-N | *O. militaris* | Celle, -, Germany | 1981 | DI |  | this study, AB701690 |
| ORM | *O. morio* | Celle, -, Germany | 1983 | (B) |  | this study, AB701692 |
| OS | *O. simia* | Celle, -, Germany | 1981 | DI |  | this study, AB701693 |
| POL 1 | *B. napus oleifera* | Poznan, -, Poland | <4.10.1993 | B |  | this study, AB701728 |
| POL 2 | *Papaver somniferum* | Czempin, -, Poland | <4.10.1993 | B |  | this study, AB701731 |
| POL 4 | *B. napus oleifera* | Grabianowo, -, Poland | <4.10.1993 | B |  | this study, AB701732 |
| PRT 1 | *B. oleracea acephala* | Madeira, -, Portugal | 1993/1994 | B |  | this study, AB701729 |
| PV0054 | *B. oleacea* | -, -, Germany | Not known | B |  | this study, AB701730 |
| PV0104 | *L. sativa* | Stuttgart, -, Germany | 1986 | BR | [6] | AB093603 |
| PV177 | *Brassica* spp. | Cambridge, UK | 1934^d^ | B |  | this study, AB701733 |
| PV376-Br | *B. napus* | Braunschweig, -, Germany | 1970 | B | [6] | AB093604 |
| Rn98 | *Ranunculus asiaticus* | -, Liguria, Italy | 1997 | B | [4] | AB252135 |
| RUS 1 | *Armoracia rusticana* | -, -, Russia | 1993 | B | [6] | AB093606 |
| RUS 2 | *B. napus* | Moscow, -, Russia | Not known | B | [6] | AB093607 |
| St48 | *Limonium sinuatum* | -, Toscana, Italy | 1993 | B | [6] | AB093596 |
| TIGA | *Tigridia* sp. | Braunschweig, -, Germany | 1983 | (B) |  | this study, AB701734 |
| TIGD | *Tigridia* sp. | Braunschweig, -, Germany | 1983 | (B) |  | this study, AB701735 |
| UK 1 | *B. napus* | -, Warwickshire, UK | 1975 | B | [21] | AF169561 |
| UT | *Utricularia* sp. | Wuerzburg, -, Germany | 1997 | B |  | this study, AB701736 |

^a^ Host type B; *Brassica*, isolates infected *B. rapa* cv. Hakatasuwari systemically giving mosaic symptoms. Host type (B); isolates infected *B. rapa* only occasionally. Host type BR; these isolates infected both *B. rapa* and *R. sativus* systemically giving mosaic symptoms. Host type B(R); isolates infected *B. rapa* systemically giving mosaic symptoms and infected *R. sativus* only occasionally.

^b^ References: [4] Ohshima *et al*. (2007); [6] Tomimura *et al*. (2003); [19] Nicolas and Laliberté (1992); [20] Ohshima *et al*. (1996); [21] Jenner *et al*. (2000); [22] Jenner *et al*. (2002); [23] Jenner *et al*. (2003); [24] Chen *et al*. (2003); [25] Suehiro *et al*. (2004); [26] Kozubek *et al.* (2007); [27] Pallett *et al*. (2007); [28] Korkmaz *et al*. (2008); [29] Farzadfar *et al*. (2009); [30] Wang *et al*. (2009).

^c^ Difficult to infect brassica plants, see Table 1.

^d^ Unclear

**Table S1.** Continued.

| Isolate | Original host | Location (City, District, Country) | Year of collection | Host type^a^ | Reference^b^ | Accession code |
| --- | --- | --- | --- | --- | --- | --- |
| Asia |  |  |  |  |  |  |
| 1J | *R. sativus* | Saga, Saga, Japan | 1977 | BR | [20] | D83184 |
| 2J | *B. pekinensis* | -, Tochigi, Japan | 1994 | BR | [6] | AB093622 |
| 59J | *R. sativus* | Saga, Saga, Japan | 1996 | BR | [6] | AB093620 |
| AD178J | *R. sativus* | Rokunohe, Aomori, Japan | 1998 | BR | [4] | AB252094 |
| AD181J | *R. sativus* | Tohoku, Aomori, Japan | 1998 | BR | [4] | AB252095 |
| AD853J | *R. sativus* | Ohhata, Aomori, Japan | 2002 | BR | [4] | AB252096 |
| AD855J | *R. sativus* | Ohminato, Aomori, Japan | 2002 | BR | [4] | AB252097 |
| AD860J | *R. sativus* | Sennai, Aomori, Japan | 2002 | BR | [4] | AB252098 |
| AKD161J | *R. sativus* | Ogachi, Akita, Japan | 1998 | BR | [4] | AB252099 |
| AKD934J | *R. sativus* | Hachiryu, Akita, Japan | 2000 | BR | [4] | AB252100 |
| AKH937J | *B. pekinensis* | Yuzawa, Akita, Japan | 2000 | BR | [4] | AB252101 |
| AT181J | *Eustoma russellianum* | Aomori, Aomori, Japan | <1998 | BR | [4] | AB252102 |
| C1 | Not known | -, Taiwan , China | Not known | Not known |  | AF394601 |
| C42J | *Brassica rapa* | Saga, Saga, Japan | 1993 | B | [6] | AB093625 |
| CH6 | *R*. *sativus* | Zengjiang, Jiangsu, China | 1999 | BR | [4] | AB252103 |
| CHK16 | *R. sativus* | Guilin, Guangxi, China | 2000 | BR | [4] | AB252104 |
| CHL13 | *R. sativus* | Lushun, Liaoning, China | 1999 | BR | [4] | AB252105 |
| CHN 1 | *Brassica* sp. | -, Taiwan, China | <1980 | BR | [6] | AB093626 |
| CHN 12 | Not known | -, -, China | <1990 | B | [22] | AY090660 |
| CHZJ26A | *B. campestris* | Jiande, Zhejiang, China | 1999 | B(R) | [4] | AB252106 |
| CP845J | *C. officinalis* | Kisarazu, Chiba, Japan | 1997 | BR | [6] | AB093614 |
| DMJ | *R. sativus* | -, Tochigi, Japan | 1996 | BR | [6] | AB093623 |
| FD27J | *R. sativus* | Fukuoka, Fukuoka, Japan | 1998 | BR | [6] | AB093618 |
| FKD001J | *R. sativus* | Sukagawa, Fukushima, Japan | 2000 | BR | [4] | AB252109 |
| FKD004J | *R. sativus* | Funehiki, Fukushima, Japan | 2000 | BR | [4] | AB252110 |
| FKH122J | *B. pekinensis* | Naraha, Fukushima, Japan | 1998 | BR | [4] | AB252111 |
| GFD462J | *R. sativus* | Yoro, Gifu, Japan | 2001 | BR | [4] | AB252115 |
| H1J | *R. sativus* | Hirosaki, Aomori, Japan | 1996 | BR | [4] | AB252118 |
| HOD517J | *R*. *sativus* | Kimobetsu, Hokkaido, Japan | 1998 | BR | [6] | AB093617 |
| HRD | *R*. *sativus* | Hongzhou, Zhejiang, China | 1998 | BR | [6] | AB093627 |
| HZ6 | *Brassica* sp. | Xiaoshan, Zhejiang, China | 1998 | B | [4] | AB252119 |
| IWD032J | *R. sativus* | Iwaizumi, Iwate, Japan | 2000 | BR | [4] | AB252120 |
| IWD038J | *R. sativus* | Yahaba, Iwate, Japan | 2000 | BR | [4] | AB252121 |
| Ka1J | *B. pekinensis* | -, Tochigi, Japan | 1994 | BR | [6] | AB093624 |
| KD32J | *R*. *sativus* | Nankan, Kumamoto, Japan | 1998 | BR | [6] | AB093621 |
| KGD54J | *R*. *sativus* | Sendai, Kagoshima, Japan | 1998 | BR | [4] | AB252123 |
| KWB778J | *B. oleracea* | Takamatsu, Kagawa, Japan | 2004 | B | [4] | AB252124 |
| KWB779J | *B. rapa* | Takamatsu, Kagawa, Japan | 2004 | BR | [4] | AB252125 |
| KYD073J | *R*. *sativus* | Mineyama, Kyoto, Japan | 2000 | BR | [4] | AB252126 |
| KYD81J | *R*. *sativus* | Joyo, Kyoto, Japan | 1998 | BR | [6] | AB093613 |
| MED302J | *R*. *sativus* | Shiroyama, Mie, Japan | 2001 | BR | [4] | AB252127 |
| MYD013J | *R*. *sativus* | Yamamoto, Miyagi, Japan | 2000 | BR | [4] | AB252128 |
| MYD015J | *R*. *sativus* | Kesennuma, Miyagi, Japan | 2000 | BR | [4] | AB252129 |
| ND10J | *R*. *sativus* | Hirato, Nagasaki, Japan | 1998 | BR | [4] | AB252130 |
| NDJ | *R*. *sativus* | Takaki, Nagasaki, Japan | 1997 | BR | [6] | AB093616 |
| NID048J | *R*. *sativus* | Niitsu, Niigata, Japan | 2000 | BR | [4] | AB252131 |
| NID119J | *R*. *sativus* | Yuzawa, Niigata, Japan | 1998 | BR | [4] | AB252132 |
| NRD350J | *R*. *sativus* | Gojyo, Nara, Japan | 2001 | BR | [4] | AB252134 |
| RC4 | *Zantedeschia* sp. | -, Taiwan, China | 2000 | BR | [24] | AY134473 |
| SGB088J | *B. rapa* | Hikone, Shiga, Japan | 2000 | BR | [4] | AB252136 |
| SGD311J | *R. sativus* | Nishiazai, Shiga, Japan | 1998 | BR | [6] | AB093619 |
| SMD060J | *R. sativus* | Gotsu, Shimane, Japan | 2000 | BR | [4] | AB252137 |
| TANX2 | *R. sativus* | Tai’an, Shandong, China | 2007 | BR | [30] | EU734433 |
| TD88J | *R*. *sativus* | Tokyo, Tokyo, Japan | 1998 | BR | [6] | AB093615 |
| TRD052J | *R. sativus* | Akasaki, Tottori, Japan | 2000 | BR | [4] | AB252138 |
| TRD053J | *R. sativus* | Tomari, Tottori, Japan | 2000 | BR | [4] | AB252139 |
| Tu-2R1 | *R. sativus* | -, Tochigi, Japan | Not known | BR | [25] | AB105135 |
| Tu-3 | *B. oleracea* | -, Tochigi, Japan | Not known | B | [25] | AB105134 |
| TW | Not known | -, Taiwan, China | Not known | Not known |  | AF394602 |
| YAD020J | *R. sativus* | Shirataka, Yamagata, Japan | 2000 | BR | [4] | AB252140 |
| YAL018J | *L. sativa* | Sakae, Yamagata, Japan | 2000 | BR | [4] | AB252141 |
| YC5 | *Zantedeschia* sp. | -, Taiwan, China | 2000 | BR | [24] | AF530055 |
| YMD069J | *R. sativus* | Misumi, Yamaguchi, Japan | 2000 | BR | [4] | AB252142 |
| YMD070J | *R. sativus* | Abu, Yamaguchi, Japan | 2000 | BR | [4] | AB252143 |
| WFLB06 | *R. sativus* | Weifang, Shandong, China | 2006 | BR | [30] | EU734434 |
|  |  |  |  |  |  |  |
| Other |  |  |  |  |  |  |
| BZ1 | *B. oleracea* | -, Federal, Brazil | 1996 | B | [6] | AB093611 |
| CDN 1 | *B. napus napobrassica* | -, -, Canada | <1988 | B | [23] | AY227024 |
| IRNTRa6 | *Rapistrum rugosum* | Varamin, Tehran, Iran | 2004 | B | [29] | AB440238 |
| IRNSS5 | *Sisymbrium loeselii* | Semnan, Semnan, Iran | 2003 | B | [29] | AB440239 |
| IS1 | *Allium ampeloprasum* | -, -, Israel | 1993 | B | [6] | AB093602 |
| KEN 1 | *B. oleracea* | -, -, Kenya | 1994 | B | [6] | AB093605 |
| NZ290 | *B. pekinensis* | -, Alan Stewart, New Zealand | 1998 | B | [6] | AB093612 |
| PV134 | *Sesynibium* sp. | -, California, USA | <1960 | B |  | this study, AB701737 |
| PV389 | *Tulipa gesnerana* | Beltsville, Maryland, USA | 1986 | B |  | this study, AB701738 |
| Q-Ca | *B. rapa* | -, -, Canada | Not known | Not known | [19] | D10927 |
| TUR1 | *B. oleracea* | Canakkale, Marmora, Turkey | 2004 | B | [28] | AB362512 |
| TUR9 | *R. sativus* | Balikesir, Marmora, Turkey | 2005 | BR | [28] | AB362513 |
| USA 1 | *B. oleracea* | -, -, USA | <1980 | B | [6] | AB093609 |
| USA 4 | *B. pekinensis* | -, -, USA | 1993 | B |  | this study, AB701739 |
| USA 5 | *R. sativus* | San Francisco, California, USA | 2002 | BR |  | this study, AB701740 |
| USA 6 | *R. sativus* | San Francisco, California, USA | 2002 | BR |  | this study, AB701741 |
