# Supplementary material for: Turnip Mosaic Potyvirus Probably First Spread to Eurasian Brassica Crops from Wild Orchids about 1000 Years Ago
Source: PLoS One. 2013 Feb 6;8(2):e55336. doi: 10.1371/journal.pone.0055336 (PMC3566190; doi:10.1371/journal.pone.0055336)
Supplement: Table S2 — Recombination sites in full genomic sequences. (DOC) [file pone.0055336.s005.doc]

**Table S2.** Recombination sites in full genomic sequences.

| Isolate | Recombinant type | Recombinant type pattern | Recombination site | Parental sequence (group) | RDP | GENECONV | BootScan | MaxChi | Chimaera | SiScan | PhylPro |
| --- | --- | --- | --- | --- | --- | --- | --- | --- | --- | --- | --- |
|  |  |  |  |  | P-value | | | | | P and Z-value |  |
| 1J | Inter- recombinant | ABR x wB3 x ABR x wB3 x wB2 | 183 | CH6(ABR) x FKH122J(wB3) | 1.15 x 10^-6^ | 2.90 x 10^-6^ | 7.23 x 10^-7^ | 4.33 x 10^-6^ | 1.11 x 10^-4^ | 1.56 x 10^-6^ |  |
|  |  |  | 750 | DNK4(wB3) x CHN1(ABR) | 2.63 x 10^-22^ | 1.98 x 10^-20^ | 1.63 x 10^-22^ | 1.55 x 10^-8^ | 2.18 x 10^-8^ | 1.54 x 10^-11^, Z>6.52 |  |
|  |  |  | 948 | YAD020J(ABR) x GBR7(wB3) | 2.38 x 10^-27^ | 8.89 x 10^-27^ | 2.32 x 10^-37^ | 1.93 x 10^-17^ | 5.30 x 10^-18^ | 1.80 x 10^-20^, Z>6.52 | Detected |
|  |  |  | 6293 | GBR36(wB3) x CDN1/Q-Ca(wB2) | 1.28 x 10^-20^ | 1.65 x 10^-11^ | 9.49 x 10^-20^ | 6.58 x 10^-8^ | 1.41 x 10^-9^ | 1.29 x 10^-13^, Z>4.2 |  |
| 2J | Intra-recombinant | wB2 x wB3 x wB3 x wB2 | 643 | PV134(wB3) x Ka1J(wB3) | 1.15 x 10^-32^ | 3.69 x 10^-19^ | 3.69 x 10^-33^ | 1.47 x 10^-6^ | 9.65 x 10^-8^ | 7.80 x 10^-14^, Z>3.98 |  |
|  |  |  | 2194 | MYD013J(wB3) x Tu2R1(wB3) | 6.03 x 10^-14^ | 4.91 x 10^-13^ | 3.58 x 10^-14^ | 6.03 x 10^-12^ | 2.96 x 10^-7^ | 2.21 x 10^-15^ |  |
|  |  |  | 6293 | GBR36(wB3) x CDN1/Q-Ca(wB2) | 1.63 x 10^-13^ | 1.39 x 10^-3^ | 9.49 x 10^-20^ | 9.56 x 10^-4^ | 8.78 x 10^-6^ | 8.37 x 10^-9^, Z>4.2 |  |
| 59J | Inter- recombinant | ABR x wB3 x ABR x wB3 | 200 | CH6(ABR) x USA4(wB3) | 1.50 x 10^-46^ | 6.76 x 10^-22^ | 1.30 x 10^-46^ | 5.72 x 10^-14^ | 3.63 x 10^-14^ | 2.67 x 10^-14^, Z>5,78 |  |
|  |  |  | 756 | USA4(wB3) x CH6(ABR) | 1.50 x 10^-46^ | 6.76 x 10^-22^ | 1.30 x 10^-46^ | 5.72 x 10^-14^ | 3.63 x 10^-14^ | 2.67 x 10^-14^, Z>6.57 |  |
|  |  |  | 9152 | CHL13(ABR) x HZ6(wB3) | 9.98 x10^-17^ | 5.69 x10^-13^ | 6.54 x10^-18^ | 1.40 x 10^-3^ | 3.71 x 10^-1^ | 1.36 x10^-4^, Z>3.44 |  |
| C42J | Intra-recombinant | wB3 x wB3 x wB2 | 739 | YAD020J(wB3) x GBR7(wB3) | 6.64 x 10^-29^ | 1.78 x 10^-16^ | 2.33 x 10^-29^ | 6.65 x 10^-16^ | 1.47 x 10^-16^ | 3.06 x 10^-15^ |  |
|  |  |  | 6300 | GBR36(wB3) x CDN1(wB2) | 3.68 x 10^-22^ | 5.71 x 10^-14^ | 1.54 x 10^-22^ | 7.84 x 10^-8^ | 8.28 x 10^-9^ | 5.03 x 10^-13^, Z>6.5 |  |
| DMJ | Intra-recombinant | wB2 x wB3 x wB3 x wB3 x wB2 | 695 | DNK2(wB2) x Ka1J(wB3) | 1.21 x 10^-22^ | 2.95 x 10^-9^ | 6.28 x 10^-23^ | 1.26 x 10^-5^ | 4.55 x 10^-6^ | 2.68 x 10^-9^ |  |
|  |  |  | 1227 | FKD004J(wB3) x HZ6(wB3) | 1.03 x 10^-12^ | 6.68 x 10^-4^ | 2.10 x 10^-12^ | 3.58 x 10^-7^ | 6.57 x 10^-9^ | 1.19 x 10^-11^, Z>4.35 |  |
|  |  |  | 2056 | HZ6(wB3) x FKD004J(wB3) | 1.03 x 10^-12^ | 6.68 x 10^-4^ | 2.10 x 10^-12^ | 3.58 x 10^-7^ | 6.57 x 10^-9^ | 1.19 x 10^-11^, Z>4.35 |  |
|  |  |  | 6293 | GBR36(wB3) x CDN1/Q-Ca(wB2) | 3.80 x 10^-21^ | 1.97 x 10^-12^ | 1.60 x 10^-21^ | 6.16 x 10^-8^ | 2.34 x 10^-9^ | 2.15 x 10^-12^, Z>4.2 |  |
| Ka1J | Intra-recombinant | wB3 x wB3 x wB2 | 748 | YAD020J(wB3) x GBR7(wB3) | 3.37 x 10^-32^ | 7.49 x 10^-23^ | 6.57 x 10^-32^ | 2.10 x 10^-14^ | 1.93 x 10^-14^ | 1.17 x 10^-16^ |  |
|  |  |  | 6293 | GBR36(wB3) x CDN1/Q-Ca(wB2) | 1.29 x 10^-16^ | 6.66 x 10^-5^ | 1.17 x 10^-16^ | 9.20 x 10^-7^ | 7.76 x 10^-8^ | 1.30 x 10^-10^, Z>4.2 |  |
| NDJ | Inter-recombinant | ABR x wB3 x ABR x wB3 | 225 | CH6(ABR) x USA4(wB3) | 2.76 x 10^-45^ | 6.70 x 10^-32^ | 2.48 x 10^-45^ | 1.55 x 10^-10^ | 4.70 x 10^-12^ | 9.55 x 10^-14^ |  |
|  |  |  | 728 | USA4(wB3) x CH6(ABR) | 2.76 x 10^-45^ | 6.70 x 10^-32^ | 2.48 x 10^-45^ | 1.55 x 10^-10^ | 4.70 x 10^-12^ | 9.55 x 10^-14^ |  |
|  |  |  | 6450 | MYD015J(ABR) x 2J(wB3) | 2.18 x10^-144^ | 3.70 x10^-137^ | 5.20 x10^-144^ | 2.68 x10^-39^ | 2.63 x 10^-28^ | 4.96 x 10^-47^ |  |
| FD27J | Inter-recombinant | ABR x wB3 x ABR x wB3 x ABR | 183 | CH6(ABR) x FKH122J(wB3) | 1.10 x 10^-8^ | 3.37 x 10^-8^ | 7.54 x 10^-9^ | 8.05 x 10^-3^ | 2.36 x 10^-3^ | 1.40 x 10^-7^, Z>6.07 |  |
|  |  |  | 750 | DNK4(wB3) x CHN1(ABR) | 7.46 x 10^-15^ | 3.71 x 10^-16^ | 5.12 x 10^-15^ | 8.69 x 10^-6^ | 8.78 x 10^-6^ | 1.67 x 10^-11^, Z>6.07 |  |
|  |  |  | 931 | HRD(ABR) x FKD004J(wB3) | 1.43 x 10^-73^ | 1.08 x 10^-64^ | 1.30 x 10^-70^ | 2.68 x 10^-35^ | 2.16 x 10^-26^ | 1.85 x 10^-33^, Z>6.07 | Detected |
|  |  |  | 2989 | FKD004J(wB3) x HRD(ABR) | 1.43 x 10^-73^ | 1.08 x 10^-64^ | 1.30 x 10^-70^ | 2.68 x 10^-35^ | 2.16 x 10^-26^ | 1.85 x 10^-33^, Z>6.07 | Detected |
| KD32J | Inter-recombinant | ABR x wB3 x ABR x wB3 | 224 | CH6(ABR) x USA4(wB3) | 2.21 x 10^-41^ | 2.45 x 10^-22^ | 3.08 x 10^-42^ | 2.92 x 10^-11^ | 6.01 x 10^-12^ | 3.97 x 10^-13^ |  |
|  |  |  | 728 | USA4(wB3) x CH6(ABR) | 2.21 x 10^-41^ | 2.45 x 10^-22^ | 3.08 x 10^-42^ | 2.92 x 10^-11^ | 6.01 x 10^-12^ | 3.97 x 10^-13^ |  |
|  |  |  | 9175 | CHL13(ABR) x HZ6(wB3) | 1.74 x 10^-15^ | 5.48 x 10^-12^ | 1.27 x 10^-16^ | 5.01 x 10^-3^ | 3.71 x 10^-1^ | 3.82 x 10^-4^, Z>3.25 |  |
| KYD81J | Intra-recombinant | bBR x bBR | 867 | TANX2(bBR) x USA6(bBR) | 8.04 x 10^-10^ | 2.81 x 10^-3^ | 2.70 x 10^-9^ | 5.01 x 10^-8^ | 3.84 x 10^-7^ | 1.33 x 10^-10^ |  |
| TD88J | Inter-recombinant | bBR x ABR x bBR x bBR | 2449 | DEU4(bBR) x ND10J(ABR) | 5.16 x10^-76^ | 3.43 x10^-66^ | 3.35 x10^-73^ | 1.41 x10^-17^ | 8.70 x10^-20^ | 3.64 x10^-22^ | Detected |
|  |  |  | 3016 | ND10J(ABR) x DEU4(bBR) | 5.16 x10^-76^ | 3.43 x10^-66^ | 3.35 x10^-73^ | 1.41 x10^-17^ | 8.70 x10^-20^ | 3.64 x10^-22^ |  |
|  |  |  | 6541 | TANX2(bBR) x NID119J(bBR) | 1.13 x10^-29^ | 2.69 x10^-17^ | 7.31 x10^-29^ | 1.66 x10^-13^ | 6.36 x10^-11^ | 3.68 x10^-18^ |  |
| CP845J | Intra-recombinant | bBR x bBR x bBR | 867 | TANX2(bBR) x USA6(bBR) | 1.60 x 10^-12^ | 4.08 x 10^-5^ | 1.12 x 10^-12^ | 6.91 x 10^-10^ | 2.82 x 10^-9^ | 1.23 x 10^-13^ |  |
|  |  |  | 1713 | ITA7(bBR) x KWB779J/PV0104(bBR) | 1.26 x 10^-9^ | 1.11 x 10^-1^ | 5.15 x 10^-10^ | 1.13 x 10^-8^ | 5.12 x 10^-10^ | 4.39 x 10^-20^, Z<3.0 |  |
| HOD517J | Inter-recombinant | wB3 x ABR x wB3 x ABR x wB3 | 756 | USA4(wB3) x CH6(ABR) | 1.14 x10^-47^ | 3.16 x10^-13^ | 2.52 x10^-47^ | 3.43 x10^-17^ | 6.81 x10^-17^ | 3.84 x10^-15^ |  |
|  |  |  | 5284 | SMD060J(ABR) x FKD001J(wB3) | 2.04 x10^-31^ | 2.63 x10^-22^ | 2.03 x10^-30^ | 1.89 x10^-7^ | 1.80 x10^-7^ | 5.35 x10^-9^ |  |
|  |  |  | 5732 | FKD001J(wB3) x SMD060J(ABR) | 2.04 x10^-31^ | 2.63 x10^-22^ | 2.03 x10^-30^ | 1.89 x10^-7^ | 1.80 x10^-7^ | 5.35 x10^-9^ | Detected |
|  |  |  | 6097 | MYD015J(ABR) x 2J(wB3) | 1.00 x10^-103^ | 2.17 x10^-51^ | 6.87 x10^-100^ | 1.70 x10^-37^ | 7.89 x10^-4^ | 1.21 x10^-44^ | Detected |
| SGD311J | Inter-recombinant | ABR x wB3 x ABR x wB3 x ABR | 190 | CH6(ABR) x USA4(wB3) | 7.19 x10^-36^ | 1.11 x10^-15^ | 1.25 x10^-35^ | 3.93 x10^-11^ | 1.41 x10^-11^ | 7.89 x10^-13^ |  |
|  |  |  | 745 | USA4(wB3) x CH6(ABR) | 7.19 x10^-36^ | 1.11 x10^-15^ | 1.25 x10^-35^ | 3.93 x10^-11^ | 1.41 x10^-11^ | 7.89 x10^-13^ |  |
|  |  |  | 5013 | SMD060J(ABR) x FKD001J(wB3) | 6.83 x10^-43^ | 9.92 x10^-30^ | 1.53 x10^-41^ | 8.59 x10^-11^ | 2.88 x10^-12^ | 5.14 x10^-15^ | Detected |
|  |  |  | 5726 | FKD001J(wB3) x SMD060J(ABR) | 6.83 x10^-43^ | 9.92 x10^-30^ | 1.53 x10^-41^ | 8.59 x10^-11^ | 2.88 x10^-12^ | 5.14 x10^-15^, Z>6.58 | Detected |
| Al | Non-recombinant | basal-B1 (bB1) |  |  |  |  |  |  |  |  |  |
| A64 | Inter-recombinant | bB1 x wB3 | 9285 | Al(bB1) x CZE1(wB3) | 8.54 x10^-10^ | 8.58 x10^-4^ | 4.74 x10^-11^ | 4.71 x10^-1^ | ND | 2.56 x10^-8^, Z>3.88 |  |
| A102/11 | Inter-recombinant | wB3/bBR x bB1 x bB1 x wB1 | 605 | Rn98(wB3/bBR?) x GRC42(bB1) | 1.23 x10^-7^ | 9.62 x10^-13^ | 1.98 x10^-8^ | 2.22 x10^-3^ | 1.42 x10^-1^ | 1.38 x10^-15^ |  |
|  |  |  | 2020 | ITA1A(bB1) x Eru1D(bB1) | 3.49 x10^-54^ | 1.10 x10^-83^ | 1.24 x10^-50^ | 6.34 x10^-38^ | 1.34 x10^-22^ | 2.88 x10^-45^ |  |
|  |  |  | 9183 | Al(bB1) x CZE1(wB1) | 2.97 x10^-10^ | 2.56 x10^-3^ | 1.28 x10^-10^ | 4.89 x10^-4^ | ND | 7.60 x10^-6^ |  |
| QCa | Non-recombinant | world-B2 (wB2) |  |  |  |  |  |  |  |  |  |
| CDN1 | Intra-recombinant | wB2 x wB3 x wB2 | 5200 | QCa(wB2) x YC5 (wB3) |  |  |  |  |  | Z>8.57 |  |
|  |  |  | 5300 | YC5 (wB3) x QCa (wB2) |  |  |  |  |  | Z>5.93 | Detected |
| CHN12 | Intra-recombinant | wB3 x wB3 x wB2 | 748 | YAD020J(wB3) x GBR7(wB3) | 4.06 x10^-33^ | 4.91 x10^-14^ | 2.51 x10^-33^ | 4.44 x10^-13^ | 8.53 x10^-15^ | 7.74 x10^-16^ |  |
|  |  |  | 6293 | GBR36(wB3) x CDN1/Q-Ca(wB2) | 5.40 x 10^-24^ | 6.79 x 10^-18^ | 6.10 x 10^-25^ | 3.68 x 10^-8^ | 5.55 x 10^-9^ | 1.41 x 10^-13^, Z>4.2 |  |
| NZ290 | Intra-recombinant | wB2 x wB2 | 7992 | DNK3(wB2) x CDN1(wB2) | 1.23 x10^-15^ | 3.79 x10^-7^ | 9.91 x10^-16^ | 5.93 x10^-7^ | 1.43 x10^-7^ | 3.53 x10^-12^ |  |
| UK1 | Intra-recombinant | wB3 x wB3 x wB2 | 738 | YAD020J(wB3) x GBR7(wB3) | 2.28 x10^-35^ | 1.46 x10^-21^ | 5.57 x10^-36^ | 5.32 x10^-15^ | 2.19 x10^-15^ | 6.67 x10^-16^ |  |
|  |  |  | 6293 | GBR36(wB3) x CDN1/Q-Ca(wB2) | 1.31 x 10^-21^ | 4.64 x 10^-12^ | 7.06 x 10^-22^ | 1.94 x 10^-8^ | 6.92 x 10^-9^ | 2.58 x 10^-13^, Z>4.2 |  |
| CHN1 | Inter-recombinant | ABR x wB3 | 8768 | MYD015J(ABR) x 2J(wB3) | 4.27 x10^-47^ | 1.14 x10^-76^ | 6.50 x10^-45^ | 1.04 x10^-34^ | 2.55 x10^-27^ | 1.97 x10^-84^ | Detected |
| HRD | Non-recombinant | Asian-BR (ABR) |  |  |  |  |  |  |  |  |  |
| KEN 1 | Intra-recombinant | wB3 x wB2 | 6293 | GBR36(wB3) x CDN1/Q-Ca(wB2) | 2.98 x 10^-16^ | 7.56 x 10^-9^ | 1.74 x 10^-16^ | 5.27 x 10^-7^ | 3.09 x 10^-7^ | 2.46 x 10^-10^, Z>4.2 |  |
| PV376Br | Intra-recombinant | wB3 x wB2 x wB2 | 7968 | DNK3(wB2) x GBR7(wB2) | 1.47 x10^-18^ | 2.04 x10^-1^ | 4.08 x10^-18^ | 4.50 x10^-8^ | ND | 2.30 x10^-9^, Z>4.95 |  |
|  |  |  | 8004 | DNK3(wB2) x CDN1(wB2) | 4.55 x10^-13^ | 5.93 x10^-2^ | 1.11 x10^-13^ | 1.30 x10^-8^ | 3.93 x10^-10^ | 3.06 x10^-10^, Z>4.91 |  |
| BZ1 | Non-recombinant | world-B2 (wB2) |  |  |  |  |  |  |  |  |  |
| Cal1 | Non-recombinant | basal-BR (bBR) |  |  |  |  |  |  |  |  |  |
| IS1 | Non-recombinant | basal-B2 (bB2) |  |  |  |  |  |  |  |  |  |
| PV0104 | Intra-recombinant | bBR x bBR x bBR | 922 | ITA7(bBR) x TANX2(bBR) | 7.98 x10^-29^ | 2.29 x10^-25^ | 8.19 x10^-30^ | 4.63 x10^-26^ | 2.70 x10^-19^ | 1.40 x10^-44^ |  |
|  |  |  | 5965 | KWB779J(bBR) x ITA7(bBR) | 6.74 x10^-17^ | 2.95 x10^-15^ | 2.93 x10^-16^ | 1.56 x10^-22^ | 4.26 x10^-19^ | 1.02 x10^-5^ |  |
| St48 | Inter-recombinant | wB3 x bB1 | 1543 | FRD1(wB3) x A64(bB1) | 7.13 x 10^-6^ | 1.03 x 10^-5^ | 3.54 x 10^-6^ | 8.73 x 10^-6^ | 3.12 x 10^-7^ | 1.36 x10^-18^, Z>6.04 |  |
| Rn98 | Inter-recombinant | wB3 x bB1 | 1543 | TANX2(bBR) x TIGD(bB1) | 2.31 x10^-16^ | ND | 1.88 x10^-13^ | 3.40 x10^-3^ | 6.20 x10^-6^ | 8.25 x10^-11^ |  |
|  |  |  | 1600 (tentative) | FRD1 x A64 | 4.77 x 10^-5^ | 4.30 x 10^-2^ | 4.56 x 10^-5^ | 1.18 x 10^-1^ | 1.31 x 10^-1^ | 1.98 x10^-16^, Z>3.8 |  |
| ITA 7 | Non-recombinant | basal-BR (bBR) |  |  |  |  |  |  |  |  |  |
| CZE 1 | Intra-recombinant | wB3 x wB2 x wB3 | 6009, | DNK2(wB3) x DNK3(wB2) | 7.67 x10^-76^ | 1.77 x10^-71^ | 1.62 x10^-73^ | 2.08 x10^-34^ | 3.35 x10^-20^ | 7.78 x10^-42^ |  |
|  |  |  | 8767 | DNK3(wB2) x DNK2(wB3) | 3.72 x10^-76^ | 8.50 x10^-72^ | 7.88 x10^-74^ | 1.01 x10^-34^ | 1.62 x10^-20^ | 2.82 x10^-42^ |  |
| RUS 1 | Non-recombinant | world-B1 (wB1) |  |  |  |  |  |  |  |  |  |
| RUS 2 | Intra-recombinant | wB3 x wB2 x wB3 | 6009 | DNK2(wB3) x DNK3(wB2) | 1.88 x10^-79^ | 3.19 x10^-74^ | 4.96 x10^-77^ | 1.47 x10^-34^ | 3.35 x10^-20^ | 3.02 x10^-42^ |  |
|  |  |  | 8791 | DNK3(wB2) x DNK2(wB3) | 9.11 x10^-80^ | 1.60 x10^-74^ | 2.40 x10^-77^ | 7.12 x10^-35^ | 1.62 x10^-20^ | 1.57 x10^-42^ |  |
| C1 | Intra-recombinant | wB3 x wB2 x wB3 x wB2 x wB2 | 4016 | USA1(wB3) x QCa(wB2) | 3.51 x10^-41^ | 3.07 x10^-35^ | 3.18 x10^-41^ | 4.80 x10^-10^ | 7.63 x10^-10^ | 5.69 x10^-11^ | Detected |
|  |  |  | 4445 | QCa(wB2) x USA1(wB3) | 1.70 x10^-41^ | 1.49 x10^-35^ | 1.54 x10^-41^ | 2.33 x10^-10^ | 9.58 x10^-10^ | 1.86 x10^-12^ | Detected |
|  |  |  | 5551 | QCa(wB2) x USA1(wB3) | 5.44 x10^-20^ | 9.56 x10^-18^ | 5.36 x10^-20^ | 8.40 x10^-3^ | 9.43 x10^-2^ | 6.81 x10^-4^ |  |
|  |  |  | 6293 | GBR36(wB3) x CDN1/Q-Ca(wB2) | 4.71 x 10^-11^ | 1.44 x 10^-2^ | 1.63 x 10^-11^ | 6.29 x 10^-7^ | 2.64 x 10^-7^ | 3.98 x 10^-13^, Z>4.2 |  |
| TW | Intra-recombinant | wB3 x wB3 x wB2 | 686 | YAD020J(wB3) x GBR7(wB3) | 4.64 x10^-28^ | 9.31 x10^-14^ | 5.32 x10^-28^ | 1.22 x10^-13^ | 9.20 x10^-15^ | 3.04 x10^-15^ |  |
|  |  |  | 6293 | GBR36(wB3) x CDN1/Q-Ca(wB2) | 2.95 x 10^-20^ | 5.26 x 10^-9^ | 1.13 x 10^-20^ | 6.54 x 10^-7^ | 3.21 x 10^-8^ | 4.85 x 10^-13^, Z>4.2 |  |
| Tu3 | Intra-recombinant | wB3 x wB3 x wB2 | 738 | YAD020J(wB3) x GBR7(wB3) | 2.78 x10^-27^ | 5.11 x10^-18^ | 1.81 x10^-27^ | 5.30 x10^-13^ | 2.59 x10^-14^ | 2.47 x10^-13^ |  |
|  |  |  | 6293 | GBR36(wB3) x CDN1/Q-Ca(wB2) | 3.07 x 10^-23^ | 1.43 x 10^-12^ | 1.38 x 10^-23^ | 1.10 x 10^-9^ | 1.22 x 10^-9^ | 5.35 x 10^-13^, Z>4.2 |  |
| Tu2-R1 | Intra-recombinant | wB3 x wB3 x wB2 | 748 | YAD020J(wB3) x GBR7(wB3) | 3.40 x10^-29^ | 1.36 x10^-19^ | 4.44 x10^-29^ | 2.01 x10^-14^ | 1.32 x10^-14^ | 7.07 x10^-16^ |  |
|  |  |  | 6293 | GBR36(wB3) x CDN1/Q-Ca(wB2) | 4.50 x 10^-14^ | 4.02 x 10^-3^ | 4.74 x 10^-14^ | 9.04 x 10^-6^ | 1.53 x 10^-7^ | 4.25 x 10^-9^, Z>4.2 |  |
| RC4 | Intra-recombinant | wB3 x wB3 x wB2 x wB2/wB3 | 747 | YAD020J(wB3) x GBR7(wB3) | 7.02 x10^-26^ | 1.21 x10^-10^ | 3.73 x10^-26^ | 4.41 x10^-13^ | 4.02 x10^-15^ | 2.56 x10^-14^ |  |
|  |  |  | 6293 | GBR36(wB3) x CDN1/Q-Ca(wB2) | 1.24 x 10^-19^ | 1.30 x 10^-9^ | 4.49 x 10^-20^ | 4.30 x 10^-8^ | 5.37 x 10^-10^ | 6.80 x 10^-13^, Z>4.2 |  |
|  |  |  | 7406-7414 | DNK2(wB3) x NLD1(wB2/wB3) | 7.12 x 10^-8^ | 1.43 x 10^-3^ | 7.88 x 10^-8^ | 5.05 x 10^-6^ | 4.05 x 10^-6^ | 2.42 x 10^-10^, Z<3.0 |  |
| YC5 | Intra-recombinant | wB3 x wB3 x wB2 | 747 | YAD020J(wB3) x GBR7(wB3) | 5.63 x10^-29^ | 1.43 x10^-16^ | 1.02 x10^-29^ | 3.85 x10^-15^ | 2.22 x10^-16^ | 2.42 x10^-14^ |  |
|  |  |  | 6293 | GBR36(wB3) x CDN1/Q-Ca(wB2) | 1.92 x 10^-22^ | 1.93 x 10^-11^ | 7.15 x 10^-23^ | 1.55 x 10^-9^ | 8.36 x 10^-10^ | 5.90 x 10^-15^, Z>4.2 |  |
| GBR 36 | Intra-recombinant | wB3 x wB3 | 746 | YAD020J(wB3) x GBR7(wB3) | 1.22 x10^-36^ | 4.11 x10^-19^ | 2.38 x10^-36^ | 3.34 x10^-15^ | 3.60 x10^-15^ | 2.47 x10^-15^ |  |
| GBR 50 | Intra-recombinant | wB3 x wB3 x wB2 | 760 | YAD020J(wB3) x GBR7(wB3) | 1.17 x10^-39^ | 7.30 x10^-26^ | 4.45 x10^-40^ | 1.61 x10^-15^ | 1.77 x10^-16^ | 1.06 x10^-16^ |  |
|  |  |  | 6293 | GBR36(wB3) x CDN1/Q-Ca(wB2) | 4.86 x 10^-16^ | 1.06 x 10^-4^ | 2.75 x 10^-16^ | 2.85 x 10^-8^ | 4.04 x 10^-8^ | 9.97 x 10^-12^, Z>4.2 |  |
| GRC 17 | Intra-recombinant | wB3 x wB3 x wB2 | 738 | YAD020J(wB3) x GBR7(wB3) | 3.59 x10^-30^ | 9.43 x10^-20^ | 1.20 x10^-30^ | 8.27 x10^-15^ | 9.47 x10^-15^ | 2.32 x10^-14^ |  |
|  |  |  | 6293 | GBR36(wB3) x CDN1/Q-Ca(wB2) | 1.23 x 10^-22^ | 6.96 x 10^-10^ | 6.81 x 10^-23^ | 2.35 x 10^-10^ | 6.02 x 10^-11^ | 9.84 x 10^-14^, Z>4.2 |  |
| GRC42 | Non-recombinant | basal-B1 (bB1) |  |  |  |  |  |  |  |  |  |
| ITA 3 | Inter-recombinant | bBR x bB2 | 644 | ITA8(bBR) x IS1(bB2) | 3.45 x10^-24^ | 1.10 x10^-16^ | 1.66 x10^-23^ | 2.67 x10^-6^ | 3.08 x10^-6^ | 3.61 x10^-17^ |  |
| DNK 2 | Intra-recombinant | wB2 x wB1 | 2453 | DNK3(wB2) x CAR37A(wB1) | 3.41 x10^-124^ | 7.55 x10^-117^ | 2.07 x10^-121^ | 1.28 x10^-39^ | 3.38 x10^-26^ | 3.71 x10^-42^ |  |
| CZE 5 | Non-recombinant | world-B2 |  |  |  |  |  |  |  |  |  |
| FRD 1 | Intra-recombinant | wB2 x wB2/wB3 x wB2 | 711 | NLD1(wB2) x DNK3(wB2) | 5.52 x 10^-16^ | 1.14 x 10^-4^ | 2.35 x 10^-16^ | 4.76 x 10^-5^ | 5.02 x 10^-7^ | 8.27 x 10^-7^, Z>4.02 |  |
|  |  |  | 6550 | GBR36(wB3) x CDN1(wB2) | 7.33 x 10^-10^ | 5.68 x 10^-7^ | 1.49 x 10^-9^ | 1.04 x 10^-4^ | 4.03 x 10^-5^ | 6.87 x 10^-12^, Z>5.17 |  |
|  |  |  | 7406-7414(tentative) | DNK2 x NLD1 | ND | ND | ND | ND | ND | Z>4.2 |  |
| NLD 1 | Intra-recombinant | wB2 x wB2 | 6293 | GBR36(wB3) x CDN1/Q-Ca(wB2) | 4.69 x 10^-17^ | 6.40 x 10^-8^ | 1.66 x 10^-17^ | 4.54 x 10^-9^ | 7.42 x 10^-10^ | 3.23 x 10^-14^, Z>4.2 |  |
| H1J | Inter-recombinant | wB3 x ABR x wB3 x wB2 x ABR x wB3 | 758 | USA4(wB3) x CH6(ABR) | 2.17 x 10^-47^ | 4.21 x 10^-27^ | 1.83 x 10^-46^ | 4.41 x 10^-17^ | 6.26 x 10^-17^ | 3.22 x 10^-16^ |  |
|  |  |  | 5207 | MYD015J(ABR) x 2J(wB3) | 2.78 x 10^-114^ | 1.80 x 10^-108^ | 5.77 x 10^-112^ | 6.56 x 10^-45^ | 2.26 x 10^-26^ | 1.80 x 10^-49^ |  |
|  |  |  | 6293 | GBR36(wB3) x CDN1/Q-Ca(wB2) | 1.23 x 10^-22^ | 6.96 x 10^-10^ | 6.81 x 10^-23^ | 2.35 x 10^-10^ | 6.02 x 10^-11^ | 9.84 x 10^-14^, Z>4.2 |  |
|  |  |  | 8788 | YAL018J(wB3) x TRD052J(ABR) | 1.34 x 10^-27^ | 1.31 x 10^-24^ | 5.66 x 10^-28^ | 5.93 x 10^-11^ | 9.34 x 10^-12^ | 3.96 x 10^-14^ | Detected |
|  |  |  | 9304 | TRD052J(ABR) x YAL018J(wB3) | 1.34 x 10^-27^ | 1.31 x 10^-24^ | 5.66 x 10^-28^ | 5.93 x 10^-11^ | 9.34 x 10^-12^ | 3.96 x 10^-14^ |  |
| AD178J | Inter-recombinant | wB3 x ABR x wB2 | 748 | GBR7(wB3) x YAD020J(ABR) | 6.43 x10^-30^ | 2.68 x10^-21^ | 1.35 x10^-29^ | 4.55 x10^-14^ | 1.07 x10^-14^ | 2.33 x10^-16^ |  |
|  |  |  | 6293 | GBR36(wB3) x CDN1/Q-Ca(wB2) | 1.17 x 10^-16^ | ND | 5.00 x 10^-17^ | 3.26 x 10^-8^ | 1.14 x 10^-8^ | 4.24 x 10^-11^, Z>4.2 |  |
| AD181J | Inter-recombinant | wB3 x ABR x wB3 x wB2 x ABR x wB3 | 756 | USA4(wB3) x CH6(ABR) | 4.01 x10^-55^ | 1.25 x10^-32^ | 3.22 x10^-49^ | 2.70 x10^-17^ | 3.80 x10^-17^ | 3.22 x10^-17^ |  |
|  |  |  | 5207 | MYD015J(ABR) x 2J(wB3) | 1.17 x10^-106^ | 3.03 x10^-101^ | 8.54 x10^-105^ | 1.83 x10^-44^ | 1.40 x10^-25^ | 3.51 x10^-47^ |  |
|  |  |  | 6293 | GBR36(wB3) x CDN1/Q-Ca(wB2) | 1.23 x 10^-22^ | 6.96 x 10^-10^ | 6.81 x 10^-23^ | 2.35 x 10^-10^ | 6.02 x 10^-11^ | 9.84 x 10^-14^, Z>4.2 |  |
|  |  |  | 8788 | YAL018J(wB3) x TRD052J(ABR) | 6.37 x10^-35^ | 1.76 x10^-32^ | 3.05 x10^-35^ | 3.01 x10^-12^ | 1.82 x10^-12^ | 3.21 x10^-15^ | Detected |
|  |  |  | 9304 | TRD052J(ABR) x YAL018J(wB3) | 6.37 x10^-35^ | 1.76 x10^-32^ | 3.05 x10^-35^ | 3.01 x10^-12^ | 1.82 x10^-12^ | 3.21 x10^-15^ |  |
| AD853J | Inter-recombinant | wB3 x ABR x wB2 | 739 | GBR7(wB3) x YAD020J(ABR) | 7.61 x10^-34^ | 1.24 x10^-22^ | 7.70 x10^-34^ | 1.55 x10^-13^ | 6.55 x10^-15^ | 9.76 x10^-16^, Z>6.41 |  |
|  |  |  | 6293 | GBR36(wB3) x CDN1/Q-Ca(wB2) | 4.85 x 10^-25^ | 4.44 x 10^-14^ | 1.99 x 10^-25^ | 6.57 x 10^-10^ | 9.73 x 10^-11^ | 2.83 x10^-15^, Z>5.99 |  |
| AD855J | Inter-recombinant | ABR x wB3 x ABR | 224 | CH6(ABR) x USA4(wB3) | 2.04 x10^-48^ | 3.63 x10^-29^ | 3.20 x10^-48^ | 6.82 x10^-13^ | 3.56 x10^-13^ | 5.99 x10^-14^ |  |
|  |  |  | 729 | HZ6(wB3) x HRD(ABR) | 1.57 x10^-64^ | 9.24 x10^-54^ | 1.45 x10^-63^ | 5.75 x10^-14^ | 6.60 x10^-15^ | 9.55 x10^-18^ |  |
| AD860J | Inter-recombinant | wB3 x ABR x wB3 x wB2 | 756 | USA4(wB3) x CH6(ABR) | 3.40 x10^-42^ | 4.38 x10^-9^ | 1.18 x10^-41^ | 1.59 x10^-16^ | 9.01 x10^-17^ | 1.82 x10^-14^ |  |
|  |  |  | 5122 | MYD015J(ABR) x 2J(wB3) | 4.50 x10^-100^ | 7.79 x10^-121^ | 6.95 x10^-99^ | 5.54 x10^-45^ | 1.04 x10^-24^ | 1.58 x10^-50^ | Detected |
|  |  |  | 6293 | GBR36(wB3) x CDN1/Q-Ca(wB2) | 1.23 x 10^-22^ | 6.96 x 10^-10^ | 6.81 x 10^-23^ | 2.35 x 10^-10^ | 6.02 x 10^-11^ | 9.84 x 10^-14^, Z>4.2 |  |
| AT181J | Inter-recombinant | wB3 x ABR x wB3 x wB2 | 756 | USA4(wB3) x CH6(ABR) | 1.52 x10^-14^ | 2.59 x10^-11^ | 9.96 x10^-14^ | 1.56 x10^-16^ | 9.01 x10^-8^ | 6.25 x10^-16^ |  |
|  |  |  | 1559 | YAD020J(ABR) x GBR7(wB3) | 3.58 x10^-100^ | 4.73 x10^-96^ | 8.21 x10^-104^ | 2.51 x10^-34^ | 1.17 x10^-23^ | 1.01 x10^-46^ | Detected |
|  |  |  | 6293 | GBR36(wB3) x CDN1/Q-Ca(wB2) | 8.70 x 10^-24^ | 2.77 x 10^-15^ | 1.76 x 10^-24^ | 1.50 x 10^-9^ | 8.01 x 10^-10^ | 1.44 x 10^-15^, Z>4.2 |  |
| AKD161J | Inter-recombinant | wB3 x ABR x wB3 | 756 | USA4(wB3) x CH6(ABR) | 3.36 x10^-41^ | 3.63 x10^-12^ | 2.58 x10^-40^ | 1.82 x10^-16^ | 4.92 x10^-17^ | 9.84 x10^-14^ |  |
|  |  |  | 5122 | MYD015J(ABR) x 2J(wB3) | 3.15 x10^-100^ | 6.03 x10^-117^ | 1.96 x10^-99^ | 1.16 x10^-44^ | 5.60 x10^-24^ | 3.09 x10^-51^ | Detected |
| AKD934J | Inter-recombinant | wB3 x ABR | 902 | FKD004J(wB3) x HRD(ABR) | 1.20 x10^-75^ | 3.38 x10^-45^ | 6.04 x10^-74^ | 7.81 x10^-22^ | 1.43 x10^-20^ | 2.05 x10^-20^ | Detected |
| AKH937J | Inter-recombinant | wB3 x ABR x wB3 x wB2 xwB2/wB3 | 756 | USA4(wB3) x CH6(ABR) | 3.68 x10^-43^ | 3.27 x10^-12^ | 1.74 x10^-42^ | 2.49 x10^-16^ | 7.01 x10^-17^ | 6.88 x10^-15^ |  |
|  |  |  | 5122 | MYD015J(ABR) x 2J(wB3) | 7.88 x10^-99^ | 3.62 x10^-115^ | 1.20 x10^-97^ | 2.88 x10^-44^ | 3.87 x10^-24^ | 3.69 x10^-50^ | Detected |
|  |  |  | 6293 | GBR36(wB3) x CDN1/Q-Ca(wB2) | 1.23 x 10^-22^ | 6.96 x 10^-10^ | 6.81 x 10^-23^ | 2.35 x 10^-10^ | 6.02 x 10^-11^ | 9.84 x 10^-14^, Z>4.2 |  |
|  |  |  | 7406-7414 | DNK2(wB2) x NLD1(wB2/wB3) | 6.04 x 10^-18^ | 1.11 x 10^-16^ | 3.94 x 10^-6^ | 1.82 x 10^-5^ | 2.12 x 10^-6^ | 3.33 x 10^-5^, Z<3.0 |  |
| IWD032J | Inter-recombinant | wB3 x ABR x wB3 | 716 | USA4(wB3) x CH6(ABR) | 1.86 x10^-49^ | 4.03 x10^-13^ | 3.95 x10^-48^ | 6.47 x10^-16^ | 5.30 x10^-15^ | 1.13 x10^-13^ |  |
|  |  |  | 5991 | MYD015J(ABR) x 2J(wB3) | 4.24 x10^-104^ | 1.08 x10^-109^ | 2.84 x10^-100^ | 2.93 x10^-42^ | 4.50 x10^-23^ | 8.14 x10^-43^ | Detected |
| IWD038J(T63) | Intra-recombinant | wB3 x wB3 x wB2 | 748 | YAD020J(wB3) x GBR7(wB3) | 6.49 x10^-31^ | 2.96 x10^-23^ | 1.59 x10^-30^ | 4.49 x10^-14^ | 3.92 x10^-15^ | 8.91 x10^-16^ |  |
|  |  |  | 6293 | GBR36(wB3) x CDN1/Q-Ca(wB2) | 9.93 x 10^-17^ | 3.56 x 10^-6^ | 5.04 x 10^-17^ | 1.90 x 10^-6^ | 6.10 x 10^-8^ | 6.49 x 10^-11^, Z>4.2 |  |
| FKD001J | Intra-recombinant | wB2/wB3 x wB3 x wB3 x wB3 x wB2 | 695 | DNK3(wB2) x Ka1J (wb3) | 3.68 x 10^-24^ | 8.58 x 10^-16^ | 1.78 x 10^-24^ | 2.55 x 10^-6^ | 1.96 x 10^-6^ | 9.88 x 10^-10^ |  |
|  |  |  | 1194 | FKD004J(wB3) x HZ6(wB3) | 5.00 x 10^-12^ | 1.51 x 10^-2^ | 8.95 x 10^-12^ | 3.14 x 10^-6^ | 1.83 x 10^-7^ | 1.00 x 10^-11^ |  |
|  |  |  | 2056 | HZ6(wB3) x FKD004J(wB3) | 1.73 x 10^-12^ | 2.12 x 10^-2^ | 3.02 x 10^-12^ | 8.82 x 10^-6^ | 6.83 x 10^-7^ | 3.54 x 10^-7^ |  |
|  |  |  | 6293 | GBR36(wB3) x CDN1/Q-Ca(wB2) | 2.27 x 10^-17^ | 1.53 x 10^-9^ | 9.36 x 10^-18^ | 4.37 x 10^-8^ | 4.74 x 10^-9^ | 1.82 x 10^-12^, Z>4.2 |  |
| FKD004J | Intra-recombinant | wB2 x wB3 x wB2 | 643 | DNK3(wB2) x Ka1J(wB3) | 8.89 x10^-24^ | 3.07 x10^-11^ | 4.98 x10^-25^ | 1.63 x10^-6^ | 4.15 x10^-7^ | 9.34 x10^-11^ |  |
|  |  |  | 6293 | GBR36(wB3) x CDN1/Q-Ca(wB2) | 3.23 x 10^-15^ | 3.02 x 10^-7^ | 1.20 x 10^-15^ | 9.15 x 10^-6^ | 4.13 x 10^-8^ | 6.81 x 10^-12^, Z>4.2 |  |
| FKH122J | Intra-recombinant | wB2 x wB3 x wB2 | 695 | DNK3(wB2) x Ka1J(wB3) | 3.87 x10^-37^ | 4.37 x10^-26^ | 2.30 x10^-37^ | 1.38 x10^-7^ | 1.54 x10^-8^ | 3.66 x10^-10^ |  |
|  |  |  | 6293 | GBR36(wB3) x CDN1/Q-Ca(wB2) | 2.89 x 10^-15^ | 1.08 x 10^-2^ | 2.57 x 10^-15^ | 7.34 x 10^-8^ | 6.84 x 10^-9^ | 1.96 x 10^-11^, Z>4.2 |  |
| MYD013J | Intra-recombinant | wB2 x wB3 x wB3 x wB2 | 643 | DNK3(wB2) x Ka1J (wb3) | 3.68 x 10^-24^ | 8.58 x 10^-16^ | 1.78 x 10^-24^ | 2.55 x 10^-6^ | 1.96 x 10^-6^ | 9.88 x 10^-10^ |  |
|  |  |  | 1194 | FKD004J(wB3) x HZ6(wB3) | 1.51 x 10^-15^ | 2.79 x 10^-5^ | 9.12 x 10^-16^ | 2.11 x 10^-8^ | 3.04 x 10^-9^ | 4.40 x 10^-14^, Z>4.35 |  |
|  |  |  | 2056 | HZ6(wB3) x FKD004J(wB3) | 2.89 x 10^-16^ | 6.66 x 10^-5^ | 1.73 x 10^-15^ | 5.81 x 10^-8^ | 6.67 x 10^-7^ | 1.02 x 10^-10^, Z>4.35 |  |
| MYD015J | Inter-recombinant | wB2 x wB3 x ABR | 701 | DNK2(wB2) x YAL018J(wB3) | 2.12 x10^-11^ | 2.83 x10^-8^ | 3.98 x10^-11^ | 1.69 x10^-8^ | 3.89 x10^-1^ | 6.60 x10^-12^, Z>5.89 |  |
|  |  |  | 1621 | FKD004J(wB3) x HRD(ABR) | 1.18 x10^-132^ | 1.16 x10^-122^ | 2.80 x10^-131^ | 2.87 x10^-34^ | 2.13 x10^-31^ | 2.40 x10^-41^ | Detected |
| YAL018J | Intra-recombinant | wB3 x wB3 x wB2 x wB2/wB3 | 748 | YAD020J(wB3) x GBR7(wB3) | 2.15 x10^-34^ | 1.69 x10^-23^ | 1.24 x10^-34^ | 2.29 x10^-15^ | 1.34 x10^-16^ | 2.19 x10^-17^ |  |
|  |  |  | 6293 | GBR36(wB3) x CDN1/Q-Ca(wB2) | 1.19 x 10^-20^ | 1.49 x 10^-8^ | 4.41 x 10^-21^ | 3.86 x 10^-8^ | 6.31 x 10^-6^ | 7.34 x 10^-14^, Z>4.2 |  |
|  |  |  | 7406-7414 | DNK2(wB2) x NLD1(wB2/wB3) | 5.28 x 10^-9^ | 7.62 x 10^-5^ | 7.46 x 10^-9^ | 5.53 x 10^-5^ | 4.28 x 10^-5^ | 2.04 x 10^-11^, Z<3.0 |  |
| YAD020J | Inter-recombinant | wB3 x ABR x wB3 | 756 | USA4(wB3) x CH6(ABR) | 2.03 x10^-42^ | 5.64 x10^-14^ | 4.38 x10^-42^ | 1.25 x10^-16^ | 3.29 x10^-17^ | 3.01 x10^-15^ |  |
|  |  |  | 5122 | MYD015J(ABR) x 2J(wB3) | 4.02 x10^-99^ | 5.97 x10^-117^ | 6.59 x10^-98^ | 1.63 x10^-44^ | 3.79 x10^-24^ | 2.93 x10^-47^ | Detected |
| NID119J | Intra-recombinant | bBR x bBR x bBR x bBR x bBR | 157 | USA6(bBR) x TANX2(bBR) | 1.03 x 10^-10^ | 2.29 x 10^-2^ | 1.59 x 10^-10^ | 1.53 x 10^-5^ | 5.06 x 10^-7^ | 2.18 x10^-10^ |  |
|  |  |  | 878 | TANX2(bBR) x USA5(bBR) | 2.72 x 10^-10^ | 1.97 x 10^-2^ | 5.01 x 10^-10^ | 1.63 x 10^-5^ | 6.49 x 10^-7^ | 2.96 x10^-10^ |  |
|  |  |  | 1713 | ITA7(bBR) x KWB779J/PV0104(bBR) | 1.94 x 10^-6^ | 1.99 x 10^-1^ | 2.45 x 10^-7^ | 4.80 x 10^-9^ | 2.17 x 10^-9^ | 5.92 x10^-18^, Z<3.0 |  |
|  |  |  | 6621 | CP845J(bBR) x DEU4(bBR) | 6.69 x 10^-37^ | 4.66 x 10^-17^ | 4.62 x 10^-36^ | 1.20 x 10^-21^ | 1.19 x 10^-17^ | 2.82 x 10^-18^ |  |
| NID048J | Inter-recombinant | ABR x wB3 x wB2 | 242 | CH6(ABR) x FKH122J(wB3) | 1.77 x 10^-26^ | 9.92 x 10^-25^ | 1.09 x 10^-26^ | 8.11 x 10^-6^ | 4.25 x 10^-6^ | 2.66 x 10^-7^ |  |
|  |  |  | 643 | PV134(wB3) x Ka1J(wB3) | 1.23 x 10^-14^ | 1.49 x 10^-10^ | 1.36 x 10^-15^ | 7.36 x 10^-3^ | 3.90 x 10^-9^ | 5.40 x 10^-9^ |  |
|  |  |  | 6293 | GBR36(wB3) x CDN1/Q-Ca(wB2) | 2.84 x 10^-22^ | 2.45 x 10^-14^ | 1.12 x 10^-22^ | 1.18 x 10^-8^ | 7.70 x 10^-11^ | 8,73 x 10^-15^, Z>4.2 |  |
| MED302J | Inter-recombinant | ABR x wB3 x ABR x wB3 | 224 | CH6(ABR) x USA4(wB3) | 2.22 x 10^-45^ | 2.49 x 10^-28^ | 7.20 x 10^-46^ | 1.02 x 10^-11^ | 1.38 x 10^-12^ | 8.89 x 10^-14^ |  |
|  |  |  | 719 | USA4(wB3) x CH6(ABR) | 2.22 x 10^-45^ | 2.49 x 10^-28^ | 7.20 x 10^-46^ | 1.02 x 10^-11^ | 1.38 x 10^-12^ | 8.89 x 10^-14^ |  |
|  |  |  | 9175 | CHL13(ABR) x HZ6(wB3) | 8.69 x 10^-13^ | 2.79 x 10^-8^ | 7.32 x 10^-14^ | ND | 9.70 x 10^-1^ | 5.77 x 10^-4^ |  |
| TRD052J | Inter-recombinant | ABR x wB3 x ABR | 224 | CH6(ABR) x USA4(wB3) | 3.47 x 10^-44^ | 8.58 x 10^-25^ | 1.15 x 10^-43^ | 3.63 x 10^-11^ | 1.72 x 10^-11^ | 1.41 x 10^-11^ |  |
|  |  |  | 728 | USA4(wB3) x CH6(ABR) | 3.47 x 10^-44^ | 8.58 x 10^-25^ | 1.15 x 10^-43^ | 3.63 x 10^-11^ | 1.72 x 10^-11^ | 1.41 x 10^-11^ |  |
| TRD053J | Inter-recombinant | ABR x wB3 x ABR x wB3 | 236 | HRD(ABR) x FKD004J(wB3) | 2.63 x 10^-36^ | 3.34 x 10^-10^ | 4.40 x 10^-36^ | 2.07 x 10^-11^ | 1.18 x 10^-10^ | 1.28 x 10^-9^ |  |
|  |  |  | 775 | FKD004J(wB3) x HRD(ABR) | 2.63 x 10^-36^ | 3.34 x 10^-10^ | 4.40 x 10^-36^ | 2.07 x 10^-11^ | 1.18 x 10^-10^ | 1.28 x 10^-9^ |  |
|  |  |  | 9175 | CHL13(ABR) x HZ6(wB3) | 2.30 x 10^-17^ | 8.10 x 10^-16^ | 1.42 x 10^-17^ | 4.95 x 10^-1^ | ND | 1.44 x 10^-4^ |  |
| SMD060J | Inter-recombinant | ABR x wB3 x ABR | 190 | CH6(ABR) x USA4(wB3) | 7.06 x 10^-47^ | 2.78 x 10^-20^ | 1.93 x 10^-46^ | 9.25 x 10^-13^ | 4.05 x 10^-12^ | 5.33 x 10^-14^ |  |
|  |  |  | 758 | USA4(wB3) x CH6(ABR) | 7.06 x 10^-47^ | 2.78 x 10^-20^ | 1.93 x 10^-46^ | 9.25 x 10^-13^ | 4.05 x 10^-12^ | 5.33 x 10^-14^ |  |
| YMD069J | Inter-recombinant | ABR x wB3 x ABR x wB3 x ABR x wB3 | 224 | CH6(ABR) x USA4(wB3) | 4.22 x 10^-38^ | 3.36 x 10^-38^ | 6.43 x 10^-11^ | 3.17 x 10^-12^ | 3.13 x 10^-12^ | 5.99 x 10^-14^ | Detected |
|  |  |  | 728 | USA4(wB3) x CH6(ABR) | 4.22 x 10^-38^ | 3.36 x 10^-38^ | 6.43 x 10^-11^ | 3.17 x 10^-12^ | 3.13 x 10^-12^ | 5.99 x 10^-14^ |  |
|  |  |  | 4996 | SMD060J(ABR) x FKD001J(wB3) | 6.84 x 10^-47^ | 1.18 x 10^-35^ | 4.06 x 10^-46^ | 9.72 x 10^-13^ | 4.39 x 10^-13^ | 2.28 x 10^-15^ | Detected |
|  |  |  | 5723 | FKD001J(wB3) x SMD060J | 6.84 x 10^-47^ | 1.18 x 10^-35^ | 4.06 x 10^-46^ | 9.72 x 10^-13^ | 4.39 x 10^-13^ | 2.28 x 10^-15^ | Detected |
|  |  |  | 6097 | MYD015J(ABR) x 2J(wB3) | 5.99 x 10^-89^ | 2.40 x 10^-76^ | 1.36 x 10^-87^ | 1.58 x 10^-36^ | 4.79 x 10^-16^ | 9.85 x 10^-42^ | Detected |
| YMD070J | Inter-recombinant | ABR x wB3 x ABR x wB3 | 239 | CH6(ABR) x USA4(wB3) | 2.26 x 10^-47^ | 3.59 x 10^-26^ | 1.01 x 10^-43^ | 2.18 x 10^-10^ | 1.41 x 10^-11^ | 3.50 x 10^-13^ |  |
|  |  |  | 728 | USA4(wB3) x CH6(ABR) | 2.26 x 10^-47^ | 3.59 x 10^-26^ | 1.01 x 10^-43^ | 2.18 x 10^-10^ | 1.41 x 10^-11^ | 3.50 x 10^-13^ |  |
|  |  |  | 9175 | CHL13(ABR) x HZ6(wB3) | 2.31 x 10^-14^ | 2.20 x 10^-11^ | 1.14 x 10^-14^ | 1.42 x 10^-3^ | 1.96 x 10^-1^ | 1.52 x 10^-3^, Z>3.08 |  |
| SGB088J | Inter-recombinant | ABR x wB3 x ABR | 214 | CH6(ABR) x USA4(wB3) | 9.77 x 10^-44^ | 1.29 x 10^-18^ | 9.79 x10^-44^ | 8.64 x 10^-13^ | 1.07 x 10^-12^ | 5.26 x 10^-14^ |  |
|  |  |  | 756 | USA4(wB3) x CH6(ABR) | 9.77 x 10^-44^ | 1.29 x 10^-18^ | 9.79 x10^-44^ | 8.64 x 10^-13^ | 1.07 x 10^-12^ | 5.26 x 10^-14^ |  |
| KYD073J | Inter-recombinant | ABR x wB3 x ABR | 224 | CH6(ABR) x USA4(wB3) | 6.53 x 10^-50^ | 1.35 x 10^-26^ | 3.00 x 10^-46^ | 1.04 x10^-10^ | 1.52 x10^-11^ | 6.80 x 10^-13^, Z>6.26 |  |
|  |  |  | 748 | USA4(wB3) x CH6(ABR) | 6.53 x 10^-50^ | 1.35 x 10^-26^ | 3.00 x 10^-46^ | 1.04 x10^-10^ | 1.52 x10^-11^ | 6.80 x 10^-13^, Z>6.95 |  |
| GFD462J | Inter-recombinant | ABR x wB3 x ABR x wB3 x ABR x bBR | 224 | CH6(ABR) x USA4(wB3) | 1.40 x 10^-42^ | 1.47 x 10^-26^ | 1.55 x 10^-42^ | 5.36 x 10^-11^ | 2.89 x 10^-12^ | 1.28 x 10^-13^ | Detected |
|  |  |  | 748 | USA4(wB3) x CH6(ABR) | 1.40 x 10^-42^ | 1.47 x 10^-26^ | 1.55 x 10^-42^ | 5.36 x 10^-11^ | 2.89 x 10^-12^ | 1.28 x 10^-13^ |  |
|  |  |  | 5509 | SMD060J(ABR) x FKD001J(wB3) | 2.30 x 10^-16^ | 5.07 x 10^-12^ | 4.65 x 10^-15^ | 5.80 x 10^-1^ | 2.15 x 10^-1^ | 1.84 x 10^-4^ | Detected |
|  |  |  | 5722 | FKD001J(wB3) x SMD060J(ABR) | 2.30 x 10^-16^ | 5.07 x 10^-12^ | 4.65 x 10^-15^ | 5.80 x 10^-1^ | 2.15 x 10^-1^ | 1.84 x 10^-4^ | Detected |
|  |  |  | 5974 | IWD032J(ABR) x CP845J(bBR) | 6.59 x 10^-186^ | 1.68 x 10^-172^ | 4.19 x 10^-191^ | 4.30 x 10^-49^ | 6.30 x 10^-31^ | 9.26 x 10^-64^ | Detected |
| NRD350J | Intra-recombinant | wB3 x wB3 x wB2 | 738 | YAD020J(wB3) x GBR7(wB3) | 1.66 x 10^-26^ | 5.44 x 10^-18^ | 5.54 x 10^-29^ | 5.65 x 10^-13^ | 7.44 x 10^-15^ | 3.73 x 10^-15^ |  |
|  |  |  | 6300 | GBR36(wB3) x CDN1(wB2) | 4.53 x 10^-22^ | 1.91 x 10^-10^ | 2.22 x 10^-22^ | 2.96 x 10^-8^ | 3.37 x 10^-9^ | 2.44 x 10^-15^, Z>5.34 |  |
| KWB778J | Intra-recombinant | wB3 x wB3 x wB2 x wB2/wB3 | 738 | YAD020J(wB3) x GBR7(wB3) | 1.15 x10^-31^ | 1.92 x10^-18^ | 2.74 x10^-32^ | 2.30 x10^-13^ | 2.74 x10^-13^ | 6.64 x10^-15^ |  |
|  |  |  | 6293 | GBR36(wB3) x CDN1/Q-Ca(wB2) | 1.21 x 10^-19^ | 2.30 x 10^-9^ | 6.37 x 10^-20^ | 3.09 x 10^-8^ | 4.77 x 10^-9^ | 1.82 x 10^-12^, Z>4.2 |  |
|  |  |  | 7406-7414 | DNK2(wB2) x NLD1(wB2/wB3) | 4.80 x 10^-8^ | 6.08 x 10^-3^ | 7.81 x 10^-8^ | 5.02 x 10^-4^ | 2.46 x 10^-4^ | 1.54 x 10^-5^, Z<3.0 |  |
| KWB779J | Non-recombinant | basal-BR (bBR) |  |  |  |  |  |  |  |  |  |
| ND10J | Inter-recombinant | ABR x wB3 x ABR x wB3 x ABR x wB3 | 190 | CH6(ABR) x USA4(wB3) | 2.05 x10^-35^ | 5.89 x10^-16^ | 1.69 x10^-37^ | 6.56 x10^-12^ | 2.43 x10^-13^ | 7.11 x10^-15^ |  |
|  |  |  | 756 | USA4(wB3) x CH6(ABR) | 2.05 x10^-35^ | 5.89 x10^-16^ | 1.69 x10^-37^ | 6.56 x10^-12^ | 2.43 x10^-13^ | 7.11 x10^-15^ |  |
|  |  |  | 4632 | SMD060J(ABR) x DMJ(wB3) | 1.21 x10^-82^ | 3.76 x10^-61^ | 5.04 x10^-82^ | 8.98 x10^-22^ | 8.13 x10^-24^ | 1.01 x10^-25^ | Detected |
|  |  |  | 5990 | DMJ(wB3) x SMD060J(ABR) | 1.21 x10^-82^ | 3.76 x10^-61^ | 5.04 x10^-82^ | 8.98 x10^-22^ | 8.13 x10^-24^ | 1.01 x10^-25^ | Detected |
| KGD54J | Inter-recombinant | ABR x wB3 x ABR x wB3 x ABR x wB3 | 184 | CH6(ABR) x USA4(wB3) | 1.85 x10^-49^ | 1.63 x10^-24^ | 4.18 x10^-43^ | 8.56 x10^-14^ | 3.21 x10^-14^ | 1.92 x10^-15^ |  |
|  |  |  | 758 | USA4(wB3) x CH6(ABR) | 1.85 x10^-49^ | 1.63 x10^-24^ | 4.18 x10^-43^ | 8.56 x10^-14^ | 3.21 x10^-14^ | 1.92 x10^-15^ |  |
|  |  |  | 4632 | SMD060J(ABR) x DMJ(wB3) | 3.15 x10^-86^ | 6.45 x10^-64^ | 1.66 x10^-86^ | 8.66 x10^-24^ | 6.30 x10^-25^ | 4.40 x10^-27^ | Detected |
|  |  |  | 6001 | DMJ(wB3) x SMD060J(ABR) | 3.15 x10^-86^ | 6.45 x10^-64^ | 1.66 x10^-86^ | 8.66 x10^-24^ | 6.30 x10^-25^ | 4.40 x10^-27^ | Detected |
| CHZJ26A | Intra-recombinant | wB3 x wB3 x wB2 | 748 | YAD020J(wB3) x GBR7(wB3) | 2.08 x10^-30^ | 4.90 x10^-16^ | 6.20 x10^-30^ | 1.19 x10^-14^ | 4.04 x10^-15^ | 4.55 x10^-14^ |  |
|  |  |  | 6293 | GBR36(wB3) x CDN1/Q-Ca(wB2) | 9.23 x 10^-27^ | 4.36 x 10^-18^ | 3.06 x 10^-27^ | 3.16 x 10^-12^ | 3.94 x 10^-12^ | 6.41 x 10^-16^, Z>4.2 |  |
| CHL13 | Inter-recombinant | ABR x wB3 x ABR x bBR x ABR | 250 | HRD(ABR) x PV376Br (wB3) | 1.20 x10^-31^ | 3.17 x10^-12^ | 4.44 x10^-31^ | 2.49 x10^-15^ | 2.38 x10^-15^ | 2.48 x10^-19^, Z>4.51 | Detected |
|  |  |  | 728 | USA4(wB3) x CH6(ABR) | 5.94 x10^-38^ | 5.51 x10^-16^ | 1.53 x10^-38^ | 8.90 x10^-11^ | 4.54 x10^-11^ | 2.52 x10^-12^ |  |
|  |  |  | 5672 | TRD052J(ABR) x TANX2(bBR) | 4.92 x10^-35^ | 1.45 x10^-33^ | 3.48 x10^-35^ | 3.80 x10^-7^ | 3.13 x10^-7^ | 9.41 x10^-11^, Z>6.9 |  |
|  |  |  | 5950 | TANX2 (bBR) x TRD052J(ABR) |  |  |  |  |  | Z>6.9 | Detected |
| CH6 | Non-recombinant | Asian-BR (ABR) |  |  |  |  |  |  |  |  |  |
| CHK16 | Inter- recombinant | wB3 x ABR x wB3 | 745 | FKD004J(wB2-wB3) x HRD(ABR) | 5.81 x10^-38^ | 3.8 x10^-8^ | 2.90 x10^-37^ | 2.94 x10^-14^ | 2.25 x10^-13^ | 7.02 x10^-11^, Z>6.76 |  |
|  |  |  | 9123 | CHL13(ABR) x HZ6(wB3) | 2.01 x10^-20^ | 7.43 x10^-13^ | 1.60 x10^-18^ | 1.90 x 10^-2^ | 8.95 x 10^-1^ | 9.22 x10^-5^, Z>3.77 |  |
| HZ6 | Intra-recombinant | wB3 x wB3 x wB2 | 672 | YAD020J(wB3) x GBR7(wB3) | 9.38 x10^-36^ | 1.73 x10^-23^ | 3.37 x10^-36^ | 1.66 x10^-13^ | 7.22 x10^-16^ | 3.68 x10^-16^ |  |
|  |  |  | 6293 | GBR36(wB3) x CDN1/Q-Ca(wB2) | 1.31 x 10^-18^ | 1.30 x 10^-7^ | 1.45 x 10^-18^ | 1.22 x 10^-8^ | 7.56 x 10^-9^ | 1.03 x 10^-12^, Z>4.2 |  |
| USA 1 | Intra-recombinant | wB3 x wB2 | 6300 | GBR36(wB3) x CDN1(wB2) | 1.25 x 10^-16^ | 1.14 x 10^-5^ | 4.29 x 10^-17^ | 2.79 x 10^-10^ | 1.33 x 10^-7^ | 1.69 x 10^-14^, Z>4.55 |  |
| CAR37 | Intra-recombinant | wB3 x wB2 x wB2/wB3 x wB2 | 243 | Ka1J(wB3) x DNK3(wB2) | 1.96 x 10^-23^ | 3.37 x 10^-13^ | 1.43 x 10^-23^ | 4.49 x 10^-7^ | 7.36 x 10^-8^ | 1.03x 10^-12^ |  |
|  |  |  | 1037 | Ka1J(wB3) x PV134(wB3) | 1.10 x 10^-19^ | 5.52 x 10^-7^ | 5.81 x 10^-20^ | 1.68 x 10^-9^ | 3.32 x 10^-11^ | 2.64 x 10^-15^ |  |
|  |  |  | 6350 | GBR36(wB3) x CDN1(wB2) | 3.00 x 10^-19^ | 4.34 x 10^-9^ | 4.39 x 10^-19^ | 3.56 x 10^-8^ | 2.69 x 10^-6^ | 1.08 x 10^-13^, Z>6.08 |  |
| CAR37A | Non-recombinant | world-B1 (wB1) |  |  |  |  |  |  |  |  |  |
| CAR39 | Intra-recombinant | wB2 x wB2 | 2641 | CAR37A(wB2) x DNK3(wB2) | 8.72 x 10^-155^ | 8.88 x 10^-149^ | 1.10 x 10^-155^ | 7.61 x 10^-45^ | 1.53 x 10^-31^ | 8.71 x 10^-54^, Z>8.92 |  |
| TUR1 | Intra-recombinant | wB2 x wB3 x wB2 | 2505 | FRA2(wB2) x KEN1(wB3) | 1.15 x 10^-57^ | 1.33 x 10^-56^ | 5.23 x 10^-56^ | 1.68 x 10^-31^ | 3.73 x 10^-18^ | 1.79 x 10^-47^, Z>6.33 | Detected |
|  |  |  | 5899 | GRC17(wB3) x PV376Br(wB2) | 9.62 x 10^-62^ | 3.25 x 10^-55^ | 1.91 x 10^-58^ | 6.95 x 10^-33^ | 2.47 x 10^-21^ | 2.07 x 10^-48^, Z>5.42 |  |
| TUR9 | Non-recombinant | Asian-BR (ABR) |  |  |  |  |  |  |  |  |  |
| IRNTRa6 | Non-recombinant | basal-B2 (bB2) |  |  |  |  |  |  |  |  |  |
| IRNSS5 | Non-recombinant | basal-B2 (bB2) |  |  |  |  |  |  |  |  |  |
| WFLB06 | Inter- recombinant | ABR x bBR x ABR x bBR x ABR | 475 | NID048J(ABR) x CP845J(bBR) | 1.19 x 10^-36^ | 6.76 x 10^-13^ | 2.57 x 10^-36^ | 3.94 x 10^-10^ | 6.49 x 10^-9^ | 3.47 x 10^-6^ |  |
|  |  |  | 4132 | CP845J(bBR) x AKD934J(ABR) | 2.20 x 10^-52^ | 2.37 x 10^-46^ | 1.70 x 10^-52^ | 4.04 x 10^-12^ | 9.23 x 10^-12^ | 3.36 x 10^-15^ | Detected |
|  |  |  | 4502 | HRD(ABR) x CP845J(bBR) | 3.33 x 10^-50^ | 9.45 x 10^-45^ | 2.34 x 10^-50^ | 8.55 x 10^-13^ | 5.97 x 10^-12^ | 2.98 x 10^-14^ | Detected |
|  |  |  | 7809 | CP845J(bBR) x HRD(ABR) | 2.09 x 10^-73^ | 8.70 x 10^-63^ | 3.68 x 10^-73^ | 2.75 x 10^-23^ | 3.77 x 10^-24^ | 1.49 x 10^-26^ |  |
| TANX2 | Intra-recombinant | bBR x bBR x bBR | 1107 | ITA7(bBR) x KWB779J(bBR) | 7.05 x 10^-38^ | 9.78 x 10^-41^ | 1.01 x 10^-42^ | 9.21 x 10^-31^ | 9.95 x 10^-13^ | 1.87 x 10^-56^ |  |
|  |  |  | 5968 | KWB779J(bBR) x ITA7(bBR) | 3.41 x 10^-38^ | 4.79 x 10^-41^ | 4.90 x 10^-43^ | 7.56 x 10^-31^ | 4.81 x 10^-13^ | 9.06 x 10^-57^ |  |
| GBR 98 | Intra-recombinant | wB3 x wB3 x wB2 | 731 | YAD020J(wB3) x GBR7(wB3) | 4.13 x 10^-37^ | 6.63 x 10^-22^ | 1.11 x 10^-36^ | 7.78 x 10^-15^ | 3.51 x 10^-15^ | 1.28 x 10^-15^ |  |
|  |  |  | 6767 | GBR51(wB3) x GBR27(wB3) | 1.66 x 10^-13^ | ND | 1.00 x 10^-13^ | 3.66 x 10^-5^ | 2.38 x 10^-5^ | 1.91 x 10^-4^, Z>3.72 | Detected |
| AllA | Inter- recombinant | wB3 x bB1 | 485 | TUR1(wB3) x TIGA(bB1) | 3.12 x 10^-16^ | 1.47 x 10^-6^ | 1.01 x 10^-15^ | 4.22 x 10^-1^ | 8.38 x10^-4^ | 1.30 x 10^-13^, Z>5.17 | Detected |
| ASP | Non-recombinant | basal-B0 (bB0) |  |  |  |  |  |  |  |  |  |
| BEL 1 | Non-recombinant | world-B0 (wB0) |  |  |  |  |  |  |  |  |  |
| DEU 1 | Non-recombinant | world-B2 (wB2) |  |  |  |  |  |  |  |  |  |
| DEU 2 | Non-recombinant | world-B2 (wB2) |  |  |  |  |  |  |  |  |  |
| DEU 4 | Intra-recombinant | bBR x bBR |  | KWB779J(bBR) x ITA7(bBR) | 2.39 x 10^-18^ | 1.13 x 10^-16^ | 2.13 x 10^-17^ | 1.20 x 10^-22^ | 4.26 x 10^-19^ | 4.22 x 10^-6^, Z>5.4 |  |
| DEU 5 | Intra-recombinant | wB2 x wB2 | 7675 | DNK3(wB2) x CDN1(wB2) | 3.75 x10^-14^ | 2.81 x10^-2^ | 3.52 x10^-13^ | 2.21 x10^-7^ | 6.47 x10^-8^ | 9.62 x10^-11^ |  |
| DEU 7 | Inter- recombinant | wB3 x bB1 | 612 | TUR1(wB3) x TIGA(bB1) | 3.84 x 10^-21^ | 7.06 x 10^-12^ | 3.53 x 10^-19^ | 6.55 x 10^-6^ | 4.63 x 10^-8^ | 2.09 x 10^-17^, Z>7.04 | Detected |
| DNK 3 | Non-recombinant | world-B2 (wB2) |  |  |  |  |  |  |  |  |  |
| DNK 4 | Intra-recombinant | wB3 x wB3 x wB2 | 744 | YAD020J(wB3) x GBR7(wB3) | 1.17 x 10^-46^ | 3.86 x 10^-41^ | 5.56 x 10^-47^ | 3.39 x 10^-15^ | 1.38 x 10^-16^ | 5.62 x 10^-21^ |  |
|  |  |  | 6300 | GBR36(wB3) x CDN1(wB2) | 1.12 x 10^-24^ | 2.60 x 10^-12^ | 1.42 x 10^-24^ | 4.63 x 10^-10^ | 4.61 x 10^-11^ | 2.60 x 10^-15^, Z>5.47 |  |
| Eru1D | Intra-recombinant | bB1 x bB1 | 5533 | A64(bB1) x Al(bB1) | 2.74 x 10^-9^ | 1.11 x 10^-2^ | 1.06 x 10^-13^ | 4.23 x 10^-10^ | 4.26 x 10^-12^ | 2.29 x 10^-12^, Z>4.84 |  |
| ESP 1 | Inter- recombinant | wB1 x bB1 | 698 | PRT1(wB1) x A64(bB1) | 3.89 x 10^-37^ | 1.24 x 10^-41^ | 8.28 x 10^-40^ | 2.18 x 10^-11^ | 3.13 x 10^-10^ | 8.33 x 10^-23^ |  |
| ESP 2 | Inter- recombinant | wB1 x bB1 | 686 | PRT1(wB1) x A64(bB1) | 3.77 x 10^-30^ | 1.03 x 10^-36^ | 1.44 x 10^-32^ | 1.30 x 10^-9^ | 7.70 x 10^-10^ | 4.40 x 10^-24^, Z>6.1 |  |
| FRA 2 | Non-recombinant | world-B2 (wB2) |  |  |  |  |  |  |  |  |  |
| GBR 7 | Intra-recombinant | wB3 x wB2 | 6300 | GBR36(wB3) x CDN1(wB2) | 1.45 x 10^-21^ | 6.92 x 10^-10^ | 4.72 x 10^-22^ | 9.66 x 10^-10^ | 1.46 x 10^-10^ | 2.44 x 10^-15^, Z>5.3 |  |
| GBR 8 | Intra-recombinant | wB2 x wB3 | 6628 | POL2(wB2) x KEN1(wB3) | 2.95 x 10^-74^ | 2.98 x10^-49^ | 4.67 x 10^-74^ | 1.91 x 10^-22^ | 6.88 x 10^-23^ | 1.44 x 10^-23^ |  |
| GBR 27 | Intra-recombinant | wB3 x wB3 x wB | 738 | YAD020J(wB3) x GBR7(wB3) | 1.50 x 10^-37^ | 1.32 x10^-24^ | 6.69 x 10^-38^ | 4.67 x 10^-16^ | 1.06 x 10^-16^ | 1.10 x 10^-15^ |  |
|  |  |  | 6200 | GBR36(wB3) x CDN1(wB2) | 1.02 x 10^-23^ | 7.32 x 10^-14^ | 4.89 x 10^-24^ | 1.30 x 10^-11^ | 2.05 x 10^-11^ | 1.58 x 10^-15^, Z>5.99 |  |
| GBR 30 | Intra-recombinant | wB3 x wB3 x wB2 | 738 | YAD020J(wB3) x GBR7(wB3) | 6.85 x 10^-41^ | 3.87 x 10^-30^ | 6.91 x 10^-41^ | 9.63 x 10^-15^ | 2.56 x 10^-16^ | 6.21 x 10^-16^ |  |
|  |  |  | 6300 | GBR36(wB3) x CDN1(wB2) | 4.14 x 10^-22^ | ND | 2.42 x 10^-22^ | 1.09 x 10^-9^ | 9.64 x 10^-11^ | 4.47 x 10^-13^, Z>6.01 |  |
| GBR 31 | Intra-recombinant | wB3 x wB3 x wB2 | 748 | YAD020J(wB3) x GBR7(wB3) | 4.04 x 10^-40^ | 2.85 x 10^-28^ | 1.32 x 10^-40^ | 2.18 x 10^-15^ | 2.92 x 10^-16^ | 1.16 x 10^-16^ |  |
|  |  |  | 6350 | GBR36(wB3) x CDN1(wB2) | 5.30 x 10^-23^ | 1.67 x 10^-14^ | 3.01 x 10^-23^ | 3.68 x 10^-8^ | 1.02 x 10^-8^ | 8.38 x 10^-13^, Z>6.08 |  |
| GBR 32 | Intra-recombinant | wB3 x wB3 x wB2 | 738 | YAD020J(wB3) x GBR7(wB3) | 6.85 x 10^-41^ | 3.87 x 10^-30^ | 6.91 x 10^-41^ | 9.63 x 10^-15^ | 2.56 x 10^-16^ | 6.21 x 10^-19^ |  |
|  |  |  | 6300 | GBR36(wB3) x CDN1(wB2) | 1.40 x 10^-22^ | 8.94 x 10^-14^ | 8.00 x 10^-23^ | 1.08 x 10^-9^ | 9.64 x 10^-11^ | 4.47 x 10^-13^, Z>6.01 |  |
| GBR 38 | Intra-recombinant | wB3 x wB3 x wB2 | 702 | YAD020J(wB3) x GBR7(wB3) | 5.35 x 10^-37^ | 2.62 x 10^-19^ | 1.59 x 10^-36^ | 1.21 x 10^-13^ | 9.08 x 10^-15^ | 5.00 x 10^-15^ |  |
|  |  |  | 6800 | GBR36(wB3) x CDN1(wB2) |  |  |  |  |  | Z>4.66 | Detected |
| GBR 51 | Intra-recombinant | wB3 x wB3 | 682 | YAD020J(wB3) x GBR7(wB3) | 2.47 x 10^-38^ | 1.55 x 10^-18^ | 2.99 x 10^-38^ | 4.95 x 10^-14^ | 1.75 x 10^-14^ | 2.87 x 10^-14^ |  |
| GBR 57 | Intra-recombinant | wB0 x wB3 | 8817 | GBR83(wB0) x NLD2(wB3) | 7.79 x 10^-33^ | 7.01 x 10^-29^ | 5.23 x 10^-34^ | 2.41 x 10^-8^ | 8.41 x 10^-5^ | 1.16 x 10^-13^ |  |
| GBR 83 | Non-recombinant | world-B0 (wB0) |  |  |  |  |  |  |  |  |  |
| GBR 91 | Intra-recombinant | wB3 x wB3 x wB2 | 748 | YAD020J(wB3) x GBR7(wB3) | 3.01 x 10^-40^ | 1.70 x 10^-28^ | 4.16 x 10^-41^ | 1.59 x 10^-14^ | 5.40 x 10^-16^ | 2.55 x 10^-16^ |  |
|  |  |  | 6300 | GBR36(wB3) x CDN1(wB2) | 1.16 x 10^-21^ | 2.26 x 10^-14^ | 6.20 x 10^-22^ | 3.03 x 10^-10^ | 4.11 x 10^-11^ | 1.69 x 10^-14^, Z>5.99 |  |
| GK1 | Non-recombinant | basal-B2 (bB2) |  |  |  |  |  |  |  |  |  |
| HUN 1 | Non-recombinant | world-B2 (wB2) |  |  |  |  |  |  |  |  |  |
| ITA1 A | Non-recombinant | basal-B1 (bB1) |  |  |  |  |  |  |  |  |  |
| ITA 2 | Non-recombinant | basal-B1 (bB1) |  |  |  |  |  |  |  |  |  |
| ITA 4 | Inter- recombinant | bBR x bB2 | 657 | ITA8(bBR) x IS1(bB2) | 4.91 x 10^-25^ | 4.15 x 10^-21^ | 4.06 x 10^-24^ | 2.95 x 10^-6^ | 5.36 x 10^-6^ | 2.57 x 10^-17^ |  |
| ITA 5 | Inter-recombinant | bBR x bB2 | 644 | ITA8(bBR) x IS1(bB2) | 2.46 x 10^-23^ | 1.09 x 10^-14^ | 1.29 x 10^-22^ | 5.25 x 10^-6^ | 3.78 x 10^-7^ | 5.88 x 10^-16^ |  |
| ITA 6 | Inter- recombinant | bBR x bB2 | 654 | ITA8(bBR) x IS1(bB2) | 2.76 x 10^-26^ | 6.94 x 10^-22^ | 5.32 x 10^-26^ | 1.57 x 10^-6^ | 5.37 x 10^-7^ | 3.18 x 10^-18^ |  |
| ITA 8 | Non-recombinant |  |  |  |  |  |  |  |  |  |  |
| ITA 9A | Intra-recombinant | bB1 x bB1 | 1106 | ITA1A(bB1) x A64(bB1) | 1.53 x10^-63^ | 3.55 x10^-62^ | 6.85 x10^-62^ | 1.54 x10^-24^ | 4.40 x10^-16^ | 1.49 x10^-36^, Z>6.22 |  |
|  |  |  | 2468 | GRC42(bB1) x A64(bB1) | 2.51 x 10^-8^ | 1.48 x 10^-7^ | 5.42 x 10^-10^ | 1.41 x 10^-10^ | 1.64 x 10^-5^ | 5.47 x 10^-32^ |  |
| NLD 2 | Intra-recombinant | wB3 x wB3 x wB2 | 738 | YAD020J(wB3) x GBR7(wB3) | 5.12 x10^-33^ | 7.49 x10^-20^ | 3.40 x10^-33^ | 2.86 x10^-14^ | 1.75 x10^-14^ | 1.66 x10^-14^ |  |
|  |  |  | 6300 | GBR36(wB3) x CDN1(wB2) | 4.42 x 10^-22^ | 1.16 x 10^-9^ | 2.29 x 10^-22^ | 4.59 x 10^-9^ | 8.16 x 10^-10^ | 3.23 x 10^-14^, Z>5.4 |  |
| OM-N | Non-recombinant | Orchis |  |  |  |  |  |  |  |  |  |
| OM-A | Non-recombinant | Orchis |  |  |  |  |  |  |  |  |  |
| ORM | Non-recombinant | Orchis |  |  |  |  |  |  |  |  |  |
| OS | Non-recombinant | Orchis |  |  |  |  |  |  |  |  |  |
| POL 1 | Non-recombinant | world-B2 (wB2) |  |  |  |  |  |  |  |  |  |
| PRT 1 | Non-recombinant | world-B0 (wB2) |  |  |  |  |  |  |  |  |  |
| PV0054 | Intra-recombinant | wB2 x wB2/wB3(?) x wB2 | 711 | DNK3(wB2) x NLD1(wB2/wB3) | 4.51 x 10^-16^ | 4.78 x 10^-6^ | 1.02 x 10^-16^ | 4.40 x 10^-5^ | 5.40 x 10^-7^ | 1.18 x 10^-6^, Z>5.5 |  |
|  |  |  | 6550 | GBR36(wB3) x CDN1(wB2) | 2.58 x 10^-10^ | 4.85 x 10^-7^ | 1.23 x 10^-10^ | 5.59 x 10^-5^ | 1.35 x 10^-4^ | 6.48 x 10^-11^, Z>5.17 |  |
| POL 2 | Non-recombinant | world-B2 (wB2) |  |  |  |  |  |  |  |  |  |
| POL 4 | Intra-recombinant | wB2 x wB2 | 6484 | NZ290(wB2) x DEU1(wB2) | 2.02 x 10^-29^ | 4.78 x 10^-26^ | 1.28 x 10^-28^ | 6.02 x 10^-18^ | 5.23 x 10^-13^ | 2.75 x 10^-20^ |  |
| PV177 | Intra-recombinant | wB3 x wB3 x wB2 | 730 | YAD020J(wB3) x GBR7(wB3) | 2.88 x 10^-32^ | 1.62 x 10^-16^ | 2.28 x 10^-32^ | 5.99 x 10^-14^ | 5.04 x 10^-14^ | 9.50 x 10^-14^ |  |
|  |  |  | 6300 | GBR36(wB3) x CDN1(wB2) | 7.87 x 10^-24^ | 2.90 x 10^-9^ | 4.22 x 10^-24^ | 6.60 x 10^-10^ | 3.61 x 10^-11^ | 9.44 x 10^-16^, Z>5.61 |  |
| TIGA | Non-recombinant | basal-B1 (bB1) |  |  |  |  |  |  |  |  |  |
| TIGD | Non-recombinant | basal-B1 (bB1) |  |  |  |  |  |  |  |  |  |
| UT | Intra-recombinant | wB2 x wB2/wB3 | 7694 | DNK3(wB2) x GBR7(wB2) | 2.58 x10^-17^ | 2.12 x10^-3^ | 4.15 x10^-17^ | 7.66 x10^-8^ | 2.43 x10^-9^ | 4.36 x10^-10^ |  |
| PV134 | Inter- recombinant | wB3 x bB1/bB2 x wB3 x wB2 | 1019 | KEN1(wB3) x AllA(bB1) | 9.93 x 10^-47^ | 2.51 x 10^-2^ | 5.19 x 10^-42^ | 5.37 x 10^-24^ | 6.92 x 10^-18^ | 1.32 x 10^-24^, Z>6.11 |  |
|  |  |  | 3340 | AllA(bB1) x GBR27(wB3) | 5.53 x 10^-48^ | 5.37 x 10^-1^ | 2.16 x 10^-43^ | 3.79 x 10^-23^ | 4.41 x 10^-18^ | 1.13 x 10^-19^, Z>6.11 | Detected |
|  |  |  | 6144 | GBR51(wB3) x DNK3(wB2) | 6.97 x 10^-10^ | ND | 1.09 x 10^-9^ | 3.44 x 10^-6^ | 4.18 x 10^-1^ | 8.04 x 10^-7^, Z>6.17 |  |
| PV389 | Intra-recombinant | wB3 x wB3 x wB2 | 730 | YAD020J(wB3) x GBR7(wB3) | 1.60 x 10^-32^ | 6.09 x 10^-18^ | 1.28 x 10^-32^ | 1.01 x 10^-13^ | 9.28 x 10^-14^ | 8.64 x 10^-14^ |  |
|  |  |  | 6300 | GBR36(wB3) x CDN1(wB2) | 3.83 x 10^-23^ | 3.50 x 10^-9^ | 2.05 x 10^-23^ | 2.43 x 10^-9^ | 2.94 x 10^-10^ | 1.88 x 10^-15^, Z>5.61 |  |
| USA 4 | Intra-recombinant | wB3 x wB2 | 1745 | GBR91(wB3) x HUN1(wB2) | 2.63 x 10^-46^ | 1.31 x 10^-10^ | 1.70 x 10^-47^ | 1.16 x 10^-19^ | 5.83 x 10^-19^ | 4.58 x 10^-20^ |  |
| USA 5 | Intra-recombinant | bBR x bBR x bBR | 3973 | CP845J(bBR) x Cal1(bBR) | 7.75 x 10^-14^ | 1.20 x 10^-3^ | 9.12 x 10^-13^ | 2.42 x 10^-12^ | 7.30 x 10^-14^ | 2.18 x 10^-17^, Z>4.46 |  |
|  |  |  | 5767 | Cal1(bBR) x CP845J(bBR) | 7.75 x 10^-14^ | 1.20 x 10^-3^ | 9.12 x 10^-13^ | 2.42 x 10^-12^ | 7.30 x 10^-14^ | 2.18 x 10^-17^, Z>4.46 |  |
| USA 6 | Intra-recombinant | bBR x bBR x bBR | 3973 | CP845J(bBR) x Cal1(bBR) | 8.81 x 10^-15^ | 7.77 x 10^-6^ | 3.67 x 10^-14^ | 2.97 x 10^-13^ | 8.97 x 10^-10^ | 7.15 x 10^-19^, Z>4.46 |  |
|  |  |  | 5774 | Cal1(bBR) x CP845J(bBR) | 8.81 x 10^-15^ | 7.77 x 10^-6^ | 3.67 x 10^-14^ | 2.97 x 10^-13^ | 8.97 x 10^-10^ | 7.15 x 10^-19^, Z>4.46 |  |

Major groups: bB; basal-B, bBR; basal-BR, wB; world-B, ABR; Asian-BR. Number followed by major groups indicates the subgroup (sublineage) in the major groups [2].
