# Supplementary material for: Turnip Mosaic Potyvirus Probably First Spread to Eurasian Brassica Crops from Wild Orchids about 1000 Years Ago
Source: PLoS One. 2013 Feb 6;8(2):e55336. doi: 10.1371/journal.pone.0055336 (PMC3566190; doi:10.1371/journal.pone.0055336)
Supplement: Table S3 — Comparisons of the lengths of the genes of Japanese yam mosaic virus (JYMV), Narcissus yellow stripe virus (NYSV), Scallion mosaic virus (ScMV) and Turnip mosaic virus (TuMV). (DOC) [file pone.0055336.s006.doc]

**Table S3.** Comparisons of the lengths of the genes of *Japanese yam mosaic virus* (JYMV), *Narcissus yellow stripe virus* (NYSV), *Scallion mosaic virus* (ScMV) and *Turnip mosaic virus* (TuMV).

|  | JYMV |  |  |  |  |  |  | TuMV |  |  |
| --- | --- | --- | --- | --- | --- | --- | --- | --- | --- | --- |
| Region/protein^a^ | j1 | mild |  | NYSV |  | ScMV |  | OM-N | Al | UK1 |
| 5’ NCR | 156^b^ | 153 |  | 127 |  | 109 |  | 130^c^ | 130 | 130 |
| P1 | 975 | 981 |  | 951 |  | 633 |  | 1086 | 1086 | 1086 |
| HC-Pro | 1374 | 1374 |  | 1374 |  | 1371 |  | 1374 | 1374 | 1374 |
| P3 | 1065 | 1065 |  | 1062 |  | 1062 |  | 1065 | 1065 | 1065 |
| 6K1 | 156 | 156 |  | 156 |  | 156 |  | 156 | 156 | 156 |
| CI | 1932 | 1932 |  | 1932 |  | 1932 |  | 1932 | 1932 | 1932 |
| 6K2 | 159 | 159 |  | 159 |  | 159 |  | 159 | 159 | 159 |
| VPg | 576 | 576 |  | 573 |  | 576 |  | 576 | 576 | 576 |
| NIa-Pro | 729 | 729 |  | 729 |  | 729 |  | 729 | 729 | 729 |
| NIb | 1554 | 1554 |  | 1551 |  | 1551 |  | 1551 | 1551 | 1551 |
| CP | 870 | 870 |  | 822 |  | 834 |  | 858 | 864 | 864 |
| 3’ NCR | 211 | 211 |  | 214 |  | 212 |  | 211 | 211 | 212 |

^a^ NCR; Non-coding region, P1; Protein 1, HC-Pro; Helper component-proteinase protein, P3; Protein 3, 6K1; 6Kda 1 protein, CI; Cylindrical inclusion protein, 6K2; 6Kda 2 protein, VPg; Genome-linked viral protein; NIa-Pro; Nuclear inclusion a-proteinase protein, NIb; Nuclear inclusion b protein, CP; Coat protein.

^b^ Nucleotide numbers in red shows different from those from TuMV.

^c^ Thirty-five nucleotide sequence used for primer is included.
