# Supplementary material for: Turnip Mosaic Potyvirus Probably First Spread to Eurasian Brassica Crops from Wild Orchids about 1000 Years Ago
Source: PLoS One. 2013 Feb 6;8(2):e55336. doi: 10.1371/journal.pone.0055336 (PMC3566190; doi:10.1371/journal.pone.0055336)
Supplement: Table S4 — Comparisons of the amino acids at the polyprotein cleavage sites of Japanese yam mosaic virus (JYMV), Narcissus yellow stripe virus (NYSV), Scallion mosaic virus (ScMV) and Turnip mosaic virus (TuMV). (DOC) [file pone.0055336.s007.doc]

**Table S4.** Comparisons of the amino acids at the polyprotein cleavage sites of *Japanese yam mosaic virus* (JYMV), *Narcissus yellow stripe virus* (NYSV), *Scallion mosaic virus* (ScMV) and *Turnip mosaic virus* (TuMV).

|  | JYMV |  |  |  |  |  |  | TuMV |  |  |
| --- | --- | --- | --- | --- | --- | --- | --- | --- | --- | --- |
| Proteins and stop codon | j1 | mild |  | NYSV |  | ScMV |  | OM-N | Al | UK1 |
| P1 / HC-Pro^a^ | ITHY/S | IVRF/A |  | MKHY/S |  | IRQF/S |  | IIHF/S^b^ | IVHF/S | IVHF/S |
| HC-Pro / P3 | YLIG/G | YLVG/G |  | YLVG/G |  | YAVG/G |  | YRVG/G | YRVG/G | YRVG/G |
| P3 / 6K1 | Q/A | Q/A |  | Q/T |  | Q/S |  | Q/A | Q/A | Q/A |
| 6K1 / CI | Q/A | Q/G |  | Q/S |  | Q/A |  | Q/T | Q/A | Q/T |
| CI / 6K2 | Q/S | Q/S |  | Q/S |  | Q/T |  | Q/S | Q/S | Q/N |
| 6K2 / VPg | E/A | E/A |  | E/A |  | E/A |  | E/A | E/A | E/A |
| VPg / NIa-Pro | E/S | E/S |  | E/S |  | E/S |  | E/S | E/S | E/S |
| NIa-Pro / NIb | Q/M | Q/M |  | Q/M |  | Q/M |  | Q/T | Q/T | Q/T |
| NIb / CP | Q/S | Q/S |  | Q/S |  | Q/A |  | Q/A | Q/A | Q/A |
| Stop codon | TAA | TAA |  | TAA |  | TGA |  | TGA | TGA | TGA |

^a^ P1; Protein 1, HC-Pro; Helper component-proteinase protein, P3; Protein 3, 6K1; 6Kda 1 protein, CI; Cylindrical inclusion protein, 6K2; 6Kda 2 protein, VPg; Genome-linked viral protein; NIa-Pro; Nuclear inclusion a-proteinase protein, NIb; Nuclear inclusion b protein, CP; Coat protein.

^b^ The amino acids between the cleavage sites were same to other TuMV isolates.
