# Supplementary material for: Turnip Mosaic Potyvirus Probably First Spread to Eurasian Brassica Crops from Wild Orchids about 1000 Years Ago
Source: PLoS One. 2013 Feb 6;8(2):e55336. doi: 10.1371/journal.pone.0055336 (PMC3566190; doi:10.1371/journal.pone.0055336)
Supplement: Table S5 — Detailed results of the Bayesian coalescent analysis. (DOC) [file pone.0055336.s008.doc]

**Table S5.** Detailed results of the Bayesian coalescent analysis.

| Model | | | Marginal likelihood | Bayes factor | TMRCA (95% HPD lower – upper) | Substitution Rate (subs/site/year) | 95% HPD Rate – lower (subs/site/year) | 95% HPD Rate – upper (subs/site/year) | Population Size | 95% HPD, Population Size (lower, upper) | Population Growth Rate | 95% HDP, Growth Rate (lower, upper) |
| --- | --- | --- | --- | --- | --- | --- | --- | --- | --- | --- | --- | --- |
| Helper component-proteinase protein (HC-Pro) | | | | | | | | | | | | |
| Strict clock | Constant Size | | -19873.560 | 1.542 | 3428 (987 – 6788) | 1.76 x 10^-4^ | 3.65 x 10^-5^ | 3.25 x 10^-4^ | 5.60 x 10^3^ | 1.32 x 10^3^, 1.11 x 10^4^ | N/A^a^ | N/A |
|  | Expansion Growth | | -19872.830 | 3.186 | 3512 (1137 – 7027) | 1.55 x 10^-4^ | 4.07 x 10^-5^ | 2.80 x 10^-4^ | 4.61 x 10^4^ | 2.03 x 10^3^, 1.24 x 10^5^ | 5.81 x 10^-2^ | 7.13 x 10^-3^, 1.25 x 10^-1^ |
|  | Exponential Growth | | -19873.990 |  | 2555 (1067 – 4734) | 1.86 x 10^-4^ | 6.53 x 10^-5^ | 3.21 x 10^-4^ | 5.02 x 10^3^ | 1.88 x 10^3^, 9.77 x 10^3^ | 9.13 x 10^-4^ | 1.41 x 10^-4^, 1.76 x 10^-3^ |
|  | Bayesian Skyline | | -19873.090 | 2.477 | 5203 (1276 – 13860) | 1.18 x 10^-4^ | 1.14 x 10^-5^ | 2.22 x 10^-4^ | 2.21 x 10^4^ | 3.77 x 10^3^, 9.58 x 10^4^ | N/A | N/A |
| Relaxed Exponential | Constant Size | | -19775.180 | 8E+42 | 819 (258 - 1643) | 1.07 x 10^-3^ | 5.82 x 10^-4^ | 1.56 x 10^-3^ | 6.76 x 10^2^ | 3.33 x 10^2^, 1.12 x 10^3^ | N/A | N/A |
|  | Expansion Growth | | -19775.340 | 7E+42 | 703 (254 - 1401) | 1.07 x 10^-3^ | 5.93 x 10^-4^ | 1.58 x 10^-3^ | 1.26 x 10^4^ | 3.26 x 10^2^, 2.85 x 10^4^ | 5.13 x 10^-1^ | 3.63 x 10^-5^, 0.13 x 10^-1^ |
|  | Exponential Growth^b^ | | -19775.120 | 9E+42 | 495 (174 - 924) | 1.13 x 10^-3^ | 6.71 x 10^-4^ | 1.65 x 10^-3^ | 7.76 x 10^2^ | 3.70 x 10^2^, 1.25 x 10^3^ | 4.44 x 10^-3^ | 9.00 x 10^-4^, 1.12 x 10^-2^ |
|  | Bayesian Skyline | | -19776.410 | 2E+42 | 1203 (224 - 3166) | 7.68 x 10^-4^ | 1.97 x 10^-4^ | 1.33 x 10^-3^ | 5.51 x 10^3^ | 5.87 x 10^2^, 2.75 x 10^4^ | N/A | N/A |
| Relaxed Lognormal | Constant Size | | -19778.470 | 3E+41 | 1232 (513 - 2248) | 4.24 x 10^-4^ | 1.74 x 10^-4^ | 6.90 x 10^-4^ | 1.77 x 10^3^ | 7.09 x 10^2^, 3.21 x 10^3^ | N/A | N/A |
|  | Expansion Growth | | -19776.520 | 2E+42 | 1173 (528 - 3438) | 3.12 x 10^-4^ | 8.36 x 10^-5^ | 5.45 x 10^-4^ | 3.98 x 10^4^ | 9.14 x 10^2^, 1.34 x 10^5^ | 1.09 x 10^-1^ | 1.73 x 10^-3^, 2.31 x 10^-1^ |
|  | Exponential Growth | | -19779.190 | 1E+41 | 1063 (479 – 1891) | 4.38 x 10^-4^ | 2.02 x 10^-4^ | 6.87 x 10^-4^ | 2.01 x 10^3^ | 8.61 x 10^2^, 3.66 x 10^3^ | 1.99 x 10^-3^ | 1.30 x 10^-4^, 4.03 x 10^-3^ |
|  | Bayesian Skyline | | -19779.210 | 1E+41 | 3708 (752 - 10744) | 1.96 x 10^-4^ | 1.66 x 10^-5^ | 3.79 x 10^-4^ | 1.72 x 10^4^ | 2.36 x 10^3^, 8.07 x 10^4^ | N/A | N/A |
| Protein 3 (P3) | | | | | | | | | | | | |
| Strict clock | Constant Size | -18067.697 | | 2.155 | 5132 (1614 – 10489) | 2.20 x 10^-4^ | 5.48 x 10^-5^ | 3.73 x 10^-4^ | 4.83 x 10^3^ | 1.41 x 10^3^, 1.01 x 10^4^ | N/A | N/A |
|  | Expansion Growth | -18066.530 | | 6.920 | 6299 (1878 – 13906) | 1.66 x 10^-4^ | 3.57 x 10^-5^ | 3.06 x 10^-4^ | 7.65 x 10^4^ | 2.74 x 10^3^, 2.37 x 10^5^ | 6.55 x 10^-2^ | 7.11 x 10^-3^, 1.39 x 10^-1^ |
|  | Exponential Growth | -18068.465 | |  | 3796 (1615 – 6674) | 2.32 x 10^-4^ | 9.25 x 10^-5^ | 3.82 x 10^-4^ | 4.11 x 10^3^ | 1.58 x 10^3^, 7.48 x 10^3^ | 5.96 x 10^-4^ | 4.36 x 10^-6^, 1.23 x 10^-3^ |
|  | Bayesian Skyline | -18066.570 | | 6.653 | 10248 (1933 – 31252) | 1.34 x 10^-4^ | 5.51 x 10^-6^ | 2.71 x 10^-4^ | 3.53 x 10^4^ | 4.41 x 10^3^, 1.74 x 10^5^ | N/A | N/A |
| Relaxed Exponential | Constant Size | -18020.298 | | 8E+20 | 1071 (279 – 2511) | 1.08 x 10^-3^ | 5.59 x 10^-4^ | 1.60 x 10^-3^ | 8.38 x 10^2^ | 3.83 x 10^2^, 1.35 x 10^3^ | N/A | N/A |
|  | Expansion Growth | -18021.032 | | 4E+20 | 1013 (230 – 2248) | 1.07 x 10^-3^ | 3.01 x 10^-4^ | 1.70 x 10^-3^ | 1.88 x 10^4^ | 3.39 x 10^2^, 4.15 x 10^4^ | 4.31 x 10^-1^ | 1.25 x 10^-5^, 0.12 x 10^1^ |
|  | Exponential Growth | -18020.515 | | 7E+20 | 612 (205– 1239) | 1.08 x 10^-3^ | 4.94 x 10^-4^ | 1.68 x 10^-3^ | 1.05 x 10^3^ | 4.37 x 10^2^, 1.91 x 10^3^ | 4.01 x 10^-3^ | 8.00 x 10^-4^, 1.01 x 10^-2^ |
|  | Bayesian Skyline | -18021.024 | | 4E+20 | 1985 (243 – 5847) | 6.82 x 10^-4^ | 3.88 x 10^-5^ | 1.31 x 10^-3^ | 9.83 x 10^3^ | 6.27 x 10^2^, 6.55 x 10^4^ | N/A | N/A |
| Relaxed Lognormal | Constant Size | -18026.949 | | 1E+18 | 3194 (1158 –5950) | 3.06 x 10^-4^ | 1.24 x 10^-4^ | 5.00 x 10^-4^ | 2.81 x 10^3^ | 1.15 x 10^3^, 5.22 x 10^3^ | N/A | N/A |
|  | Expansion Growth | -18026.731 | | 1E+18 | 4639 (1297 – 10290) | 2.25 x 10^-4^ | 5.32 x 10^-5^ | 3.99 x 10^-4^ | 5.96 x 10^4^ | 1.35 x 10^3^, 1.87 x 10^5^ | 8.05 x 10^-2^ | 8.83 x 10^-3^, 1.65 x 10^-1^ |
|  | Exponential Growth | -18026.664 | | 1E+18 | 2434 (962 – 4499) | 3.26 x 10^-4^ | 1.40 x 10^-4^ | 5.14 x 10^-4^ | 2.87 x 10^3^ | 1.24 x 10^3^, 5.35 x 10^3^ | 9.04 x 10^-4^ | 1.0 x 10^-1^, 2.09 x 10^-3^ |
|  | Bayesian Skyline | -18026.312 | | 2E+18 | 8552 (1179 – 27913) | 1.74 x 10^-4^ | 7.78 x 10^-6^ | 3.62 x 10^-4^ | 3.00 x 10^4^ | 3.26 x 10^3^, 1.60 x 10^5^ | N/A | N/A |
| Nuclear inclusion b protein (NIb) | | | | | | | | | | | | |
| Strict clock | Constant Size | -21182.111 | |  | 2257 (1387 – 3306) | 2.51 x 10^-4^ | 1.55 x 10^-4^ | 3.47 x 10^-4^ | 2.51 x 10^3^ | 1.49 x 10^3^, 3.77 x 10^3^ | N/A | N/A |
|  | Expansion Growth | -21179.868 | | 9.424 | 2846 (1502 – 4623) | 2.05 x 10^-4^ | 1.05 x 10^-4^ | 3.05 x 10^-4^ | 5.72 x 10^4^ | 5.33 x 10^3^, 1.50 x 10^5^ | 7.92 x 10^-2^ | 2.85 x 10^-2^, 1.37 x 10^-1^ |
|  | Exponential Growth | -21181.779 | | 1.394 | 2074 (1275 – 3073) | 2.56 x 10^-4^ | 1.60 x 10^-4^ | 3.62 x 10^-4^ | 2.83 x 10^3^ | 1.59 x 10^3^, 4.31 x 10^3^ | 1.28 x 10^-3^ | 3.97 x 10^-4^, 2.24 x 10^-3^ |
|  | Bayesian Skyline | -21179.648 | | 11.734 | 4068 (1581 – 7632) | 1.63 x 10^-4^ | 5.27 x 10^-5^ | 2.66 x 10^-4^ | 1.52 x 10^4^ | 3.61 x 10^3^, 4.94 x 10^4^ | N/A | N/A |
| Relaxed Exponential | Constant Size | -21157.000 | | 8E+10 | 1330 (342 – 2920) | 7.04 x 10^-4^ | 3.73 x 10^-4^ | 1.00 x 10^-3^ | 1.04 x 10^3^ | 5.39 x 10^2^, 1.71 x 10^3^ | N/A | N/A |
|  | Expansion Growth | -21157.376 | | 6E+10 | 1157 (273 – 2579) | 6.46 x 10^-4^ | 2.76 x 10^-4^ | 1.07 x 10^-3^ | 6.58 x 10^4^ | 4.37 x 10^2^, 2.13 x 10^5^ | 1.46 x 10^-1^ | 3.63 x 10^-3^, 3.27 x 10^-1^ |
|  | Exponential Growth | -21157.492 | | 5E+10 | 475 (184 – 875) | 7.60 x 10^-4^ | 4.06 x 10^-4^ | 1.13 x 10^-3^ | 1.36 x 10^3^ | 6.56 x 10^2^, 2.23 x 10^3^ | 7.01 x 10^-3^ | 6.27 x 10^-4^, 1.53 x 10^-2^ |
|  | Bayesian Skyline | -21158.010 | | 3E+10 | 1058 (240 – 2431) | 6.20 x 10^-4^ | 2.28 x 10^-4^ | 9.82 x 10^-4^ | 8.53 x 10^3^ | 9.77 x 10^2^, 4.35 x 10^4^ | N/A | N/A |
| Relaxed Lognormal | Constant Size | -21162.639 | | 3E+08 | 2195 (1161 – 3494) | 2.71 x 10^-4^ | 1.52 x 10^-4^ | 3.87 x 10^-4^ | 2.36 x 10^3^ | 1.31 x 10^3^, 3.76 x 10^3^ | N/A | N/A |
|  | Expansion Growth | -21164.348 | | 5E+07 | 2794 (1209 – 4855) | 2.18 x 10^-4^ | 1.01 x 10^-4^ | 3.42 x 10^-4^ | 5.68 x 10^4^ | 3.65 x 10^3^, 1.56 x 10^5^ | 8.30 x 10^-2^ | 2.49 x 10^-2^, 1.48 x 10^-1^ |
|  | Exponential Growth | -21162.878 | | 2E+08 | 1833 (906 – 2936) | 2.76 x 10^-4^ | 1.51 x 10^-4^ | 4.05 x 10^-4^ | 2.72 x 10^3^ | 1.41 x 10^3^, 4.49 x 10^3^ | 1.54 x 10^-3^ | 4.11 x 10^-4^, 2.96 x 10^-3^ |
|  | Bayesian Skyline | -21165.873 | | 1E+07 | 4084 (1440 – 8034) | 1.67 x 10^-4^ | 5.14 x 10^-5^ | 2.80 x 10^-4^ | 9.65 x 10^3^ | 1.93 x 10^3^, 3.42 x 10^4^ | N/A | N/A |
| Coherently-evolving coat protein region (cCP) | | | | | | | | | | | | |
| Strict clock | Constant Size | -6988.608 | | 1.792 | 1069 (569–1704) | 2.57 x 10^-4^ | 1.36 x 10^-4^ | 3.73 x 10^-4^ | 1.18 x 10^3^ | 6.17 x 10^2^, 1.95 x 10^3^ | N/A | N/A |
|  | Expansion Growth | -7021.757 | | 3.671 | 1723 (539–3188) | 2.02 x 10^-4^ | 5.77 x 10^-5^ | 3.45 x 10^-4^ | 3.25 x 10^4^ | 6.04 x 10^2^, 8.93 x 10^4^ | 8.23 x 10^-2^ | 2.69 x 10^-4^, 0.22 x 10^-1^ |
|  | Exponential Growth | -6987.824 | |  | 932 (507–1482) | 2.55 x 10^-4^ | 1.35 x 10^-4^ | 3.79 x 10^-4^ | 1.48 x 10^3^ | 7.36 x 10^2^, 2.49 x 10^3^ | 3.18 x 10^-3^ | 8.81 x 10^-4^, 5.75 x 10^-3^ |
|  | Bayesian Skyline | -7189.656 | | 4.401 | 2765 (561–7333) | 1.66 x 10^-4^ | 2.66 x 10^-6^ | 2.83 x 10^-4^ | 7.37 x 10^3^ | 8.53 x 10^2^, 4.39 x 10^4^ | N/A | N/A |
| Relaxed Exponential | Constant Size | -6857.122 | | 3E+16 | 672 (179–1450) | 5.88 x 10^-4^ | 2.25 x 10^-4^ | 8.46 x 10^-4^ | 5.35 x 10^2^ | 2.79 x 10^2^, 8.39 x 10^2^ | N/A | N/A |
|  | Expansion Growth | -6866.461 | | 5E+16 | 624 (155– 1365) | 5.42 x 10^-4^ | 2.21 x 10^-4^ | 8.66 x 10^-4^ | 2.57 x 10^4^ | 2.09 x 10^2^, 8.35 x 10^4^ | 3.41 x 10^-1^ | 5.32 x 10^-5^, 0.13 x 10^1^ |
|  | Exponential Growth | -6850.188 | | 6E+16 | 271 (127–470) | 6.12 x 10^-4^ | 3.41 x 10^-4^ | 8.93 x 10^-4^ | 7.94 x 10^2^ | 3.63 x 10^2^, 1.31 x 10^3^ | 1.13 x 10^-2^ | 1.18 x 10^-3^, 2.26 x 10^-2^ |
|  | Bayesian Skyline | -6877.483 | | 5E+15 | 603 (110–1730) | 6.84 x 10^-4^ | 6.49 x 10^-5^ | 1.06 x 10^-3^ | 4.25 x 10^3^ | 3.16 x 10^2^, 2.31 x 10^4^ | N/A | N/A |
| Relaxed Lognormal | Constant Size | -6918.626 | | 4E+09 | 866 (295–1534) | 3.74 x 10^-4^ | 2.04 x 10^-4^ | 5.41 x 10^-4^ | 8.03 x 10^2^ | 4.30 x 10^2^, 1.26 x 10^3^ | N/A | N/A |
|  | Expansion Growth | -6947.521 | | 2E+09 | 1116 (318–2250) | 2.96 x 10^-4^ | 1.04 x 10^-4^ | 4.91 x 10^-4^ | 2.96 x 10^4^ | 4.14 x 10^2^, 1.07 x 10^5^ | 1.24 x 10^-1^ | 4.00 x 10^-3^, 3.01 x 10^-1^ |
|  | Exponential Growth | -6914.409 | | 4E+09 | 492 (193–884) | 3.80 x 10^-4^ | 1.96 x 10^-4^ | 5.73 x 10^-4^ | 1.11 x 10^3^ | 5.18 x 10^2^, 1.85 x 10^3^ | 6.74 x 10^-3^ | 1.12 x 10^-3^, 1.33 x 10^-2^ |
|  | Bayesian Skyline | -7060.160 | | 2E+09 | 2431 (229–7687) | 2.14 x 10^-4^ | 4.21 x 10^-6^ | 3.98 x 10^-4^ | 9.95 x 10^3^ | 7.03 x 10^2^, 7.20 x 10^4^ | N/A | N/A |
| Coherently-evolving coat protein region + 16 codons (cCP+16) | | | | | | | | | | | | |
| Strict clock | Constant Size | -7040.533 | | 4.489 | 5188 (786 – 13623) | 1.00 x 10^-4^ | 1.85 x 10^-6^ | 2.00 x 10^-4^ | 7.00 x 10^3^ | 1.00 x 10^3^, 1.87 x 10^4^ | N/A | N/A |
|  | Expansion Growth | -7041.444 | | 1.806 | 6917 (1146 – 18685) | 6.57 x 10^-5^ | 3.58 x 10^-6^ | 1.38 x 10^-4^ | 1.08 x 10^5^ | 2.56 x 10^3^, 3.46 x 10^5^ | 3.26 x 10^-2^ | 5.25 x 10^-4^, 8.18 x 10^-2^ |
|  | Exponential Growth | -7042.035 | |  | 2767 (630 – 6914) | 1.14 x 10^-4^ | 1.63 x 10^-5^ | 2.19 x 10^-4^ | 5.40 x 10^3^ | 1.09 x 10^3^, 1.38 x 10^4^ | 1.57 x 10^-3^ | 1.81 x 10^-4^, 3.19 x 10^-3^ |
|  | Bayesian Skyline | -7040.704 | | 3.785 | 12891 (1208 – 41307) | 4.13 x 10^-5^ | 2.37 x 10^-6^ | 1.03 x 10^-4^ | 6.96 x 10^4^ | 7.65 x 10^3^, 2.90 x 10^5^ | N/A | N/A |
| Relaxed Exponential | Constant Size | -6999.220 | | 4E+18 | 1122 (227 – 2597) | 4.39 x 10^-4^ | 1.80 x 10^-4^ | 7.04 x 10^-4^ | 9.44 x 10^2^ | 3.69 x 10^2^, 1.75 x 10^3^ | N/A | N/A |
|  | Expansion Growth | -6994.719 | | 4E+20 | 4070 (335 – 11643) | 1.14 x 10^-4^ | 4.98 x 10^-6^ | 2.50 x 10^-4^ | 2.83 x 10^6^ | 1.57 x 10^3^, 1.04 x 10^7^ | 7.49 x 10^-2^ | 4.13 x 10^-3^, 1.63 x 10^-1^ |
|  | Exponential Growth | -6999.261 | | 4E+18 | 371 (133 – 717) | 4.29 x 10^-4^ | 1.44 x 10^-4^ | 7.24 x 10^-4^ | 1.83 x 10^3^ | 5.37 x 10^2^, 3.90 x 10^3^ | 1.16 x 10^-2^ | 2.10 x 10^-3^, 2.25 x 10^-2^ |
|  | Bayesian Skyline^d^ | -6994.360 | | 5E+20 | 4497 (347 – 14184) | 1.04 x 10^-4^ | 7.15 x 10^-6^ | 2.30 x 10^-4^ | 7.60 x 10^4^ | 7.10 x 10^3^, 3.02 x 10^5^ | N/A | N/A |
| Relaxed Lognormal | Constant Size | -7018.724 | | 1E+10 | 2442 (379 – 5458) | 1.98 x 10^-4^ | 2.59 x 10^-5^ | 3.74 x 10^-4^ | 2.76 x 10^3^ | 5.49 x 10^2^, 5.98 x 10^3^ | N/A | N/A |
|  | Expansion Growth | -7014.287 | | 1E+12 | 6441 (592 – 17548) | 8.17 x 10^-5^ | 3.73 x 10^-6^ | 1.79 x 10^-4^ | 1.84 x 10^5^ | 1.40 x 10^3^, 6.13 x 10^5^ | 4.32 x 10^-2^ | 1.56 x 10^-3^, 9.87 x 10^-2^ |
|  | Exponential Growth | -7017.211 | | 6E+10 | 1161 (233 – 2629) | 1.93 x 10^-4^ | 3.84 x 10^-5^ | 3.62 x 10^-4^ | 3.55 x 10^3^ | 9.14 x 10^2^, 7.91 x 10^3^ | 4.19 x 10^-3^ | 1.69 x 10^-4^, 8.69 x 10^-3^ |
|  | Bayesian Skyline | -7010.831 | | 4E+13 | 9767 (571 – 32075) | 5.72 x 10^-5^ | 2.49 x 10^-6^ | 1.42 x 10^-4^ | 6.61 x 10^4^ | 6.83 x 10^3^, 2.91 x 10^5^ | N/A | N/A |

^a^ Not applicable.

^b^ Exponential-growth model (estimates for HC-Pro) is not much better than the constant-size model. So we should favour the simpler model (constant population size).

^c^ Bayesian Skyline (estimates for cCP+16); clearly the uncorrelated exponential relaxed clock is the best of the three different clock models. However, the expansion model is possibly better than the Bayesian skyline model. This is because the Bayes factor supporting the Bayesian skyline over the expansion model is not very large, and also because the expansion model has far fewer parameters. Given that there is not strong evidence to favour the Bayesian skyline, it is better to choose the simpler model (expansion).

Where models are compared the best-fit model is shaded grey.

The Logistic Growth model was not applicable to the data analysed in this study because the parameter estimates did not converge to reliable values during the computation (data not shown).
